# Impact of geographic origin on access to therapy and therapy outcomes in the Swiss Hepatitis C Cohort Study

**DOI:** 10.1371/journal.pone.0218706

**Published:** 2019-06-24

**Authors:** Matteo Brezzi, Barbara Bertisch, Maroussia Roelens, Darius Moradpour, Benedetta Terziroli Beretta-Piccoli, Nasser Semmo, Beat Müllhaupt, David Semela, Francesco Negro, Olivia Keiser, for the Swiss Hepatitis C Cohort Study

**Affiliations:** 1 Institute of Global Health, University of Geneva, Geneva, Switzerland; 2 Division of Gastroenterology and Hepatology, University Hospital Lausanne, Lausanne, Switzerland; 3 Fondazione Epatocentro Ticino, Lugano, Switzerland; 4 Department for BioMedical Research, Hepatology, University of Bern, Bern, Switzerland; 5 Swiss Hepato-Pancreato-Biliary Center and Department of Gastroenterology and Hepatology, University Hospital Zürich, Zürich, Switzerland; 6 Division of Gastroenterology and Hepatology, Cantonal Hospital St. Gallen, St. Gallen, Switzerland; 7 Divisions of Gastroenterology and Hepatology and of Clinical Pathology, University Hospitals Geneva, Geneva, Switzerland; Centers for Disease Control and Prevention, UNITED STATES

## Abstract

Late diagnosis and treatment may increase morbidity and mortality among persons with hepatitis C virus (HCV) infection. We included all participants of the Swiss Hepatitis C Cohort Study (SCCS). We used unadjusted and adjusted logistic and Cox regressions to determine the association between the geographic origin of the participants and the following outcomes: antiviral treatment status; sustained virologic response; cirrhosis at enrolment; incident cirrhosis; loss to follow-up (LTFU); and mortality. The analyses were adjusted for sex, baseline age, education, source of income, alcohol consumption, injection drug use (IDU), HCV genotype, HIV or HBV coinfection, duration of HCV infection, time since enrolment, cirrhosis, (type of) HCV treatment, and centre at enrolment. Among 5,356 persons, 1,752 (32.7%) were foreign-born. IDU was more common among Swiss- (64.1%) than foreign-born (36.6%) persons. Cirrhosis at enrolment was more frequent among foreign- than Swiss-born persons, reflecting the high frequency of cirrhosis among Italian-born persons who acquired HCV between 1950 and 1970 in Italian healthcare settings. Although antiviral treatment coverage was similar, the sustained viral response rate was increased and the mortality was lower among foreign-vs. Swiss-born persons, with the lowest mortality in persons from Asia/Oceania. LTFU was more frequent in persons from Germany, Eastern and Southern Europe, and the Americas. In conclusion, in Switzerland, a country with universal healthcare, geographic origin had no influence on hepatitis C treatment access, and the better treatment outcomes among foreign-born persons were likely explained by their lower prevalence of IDU and alcohol consumption than among Swiss-born persons.

## Introduction

Hepatitis C virus (HCV) treatment can induce sustained virologic response that reverses progression of liver disease and increases life expectancy [1–3]. Access and adherence to treatment have been associated with socio-demographic factors, including ethnicity [4–6]. Many resource-rich countries have increasing numbers of residents whose geographic origin differs from their country of residence. Little is known about access to therapy and therapy outcomes among patients with hepatitis C in these countries. In Switzerland, foreign-born residents make up 29.7% of the adult population (Swiss Federal Statistical Office, 2017). Switzerland has a firm commitment to provide equal access to care for all [7]. The rollout of HCV treatment offers the opportunity to assess whether Swiss- and foreign-born persons have equal access to care and treatment, or whether disparities exist.

HCV infections may have affected Swiss- and foreign-born persons by different transmission pathways and at different times in the person’s life. In Switzerland, the main source of HCV infection is injection drug use (IDU), which peaked in the 1980s and early 1990s [8,9]. Globally, most HCV infections have been acquired via unsafe medical procedures [10,11]. The epidemic of HCV infections in Italy and Spain in the years 1950 to 1970 [12–14] became apparent in Switzerland through an increased HCV prevalence among persons aged 60 or older who originated from these countries [15]. A previous SCCS study showed that foreign-born Swiss residents who were employed were more likely to have sustained virologic response than other groups [16].

We wanted to determine whether foreign-born and Swiss-born HCV-infected persons had different levels of access to treatment and treatment outcomes, based on data from the large Swiss Hepatitis C Cohort Study (SCCS).

## Methods

### Swiss Hepatitis C Cohort Study (SCCS)

The SCCS, established in 2000, includes persons residing in Switzerland and ≥18 years old who have anti-HCV antibodies [17]. Study sites include all five university hospitals, three large non-university hospitals, and some affiliated centres [17]. Data on demographic characteristics, HCV risk factors and genotype, progression of liver disease, HCV treatment, and concomitant infections like HBV and HIV are collected by standardized questionnaires that are completed by physicians or trained study nurses during clinical visits at enrolment and annual follow-up visits. The ethics committees of all participating centres granted ethical approval for the study and all participants gave written informed consent.

### Eligibility criteria, definitions and statistical analyses

We included everyone registered in the SCCS (up to 10 September 2017) and considered the following outcomes: a) antiviral treatment status (ATS), indicating if a person was ever treated for HCV; b) sustained virologic response (SVR); c) cirrhosis at enrolment (CAE; yes/no); d) incident cirrhosis during follow-up (IC); e) loss to follow-up (LTFU); and f) mortality. Since some persons LTFU may have died, we also analysed attrition by combining mortality and LTFU into a single outcome. We defined SVR as an undetectable HCV RNA in serum ≥12 weeks after treatment end, excluding persons where the viral RNA was only tested less than 12 weeks after treatment end. When we analysed incident cirrhosis, we included only those without cirrhosis at enrolment. People were LTFU if there was no contact for two or more years, so we only included persons who were enrolled for at least two years before the study closed. LTFU contained the following dropout categories: moving to a foreign country; wanting to discontinue; care continued in a non-SCCS centre; address change without notice; no reply to written invitations; other. Information on mortality was based on information from the centres who systematically traced all patients LTFU. No linkage with a death registry was done. If we did not know the date of death, we used the last date we knew the person was alive.

We used unadjusted and adjusted logistic regression for outcomes a-c, and Cox proportional hazard regression models for time-to-event outcomes d-f. Persons were followed from enrolment into the cohort until the endpoint occurred or until the most recent follow-up visit. We censored the study ten years after enrolment into the SCCS because most people had been followed for less time.

The main exposure of interest was geographic origin of the persons; we used country of birth as a proxy of origin and divided the participants into Swiss-born and foreign-born. In a second analysis, we divided participants into groups of at least 100 members, based on the UN classification for geographic regions [18]: Switzerland, Germany, Italy, Portugal, Southern Europe (except Italy and Portugal), Eastern Europe, Western and Northern Europe (except Germany), Asia and Oceania, Africa, and the Americas (North- and South America combined).

For the adjusted models, we included explanatory variables documented at enrolment into the SCCS: sex; age (18–40, 41–60, ≥61 years); centre (Basel, Bern, Geneva, Lausanne, Lugano, Neuchâtel, St. Gallen, Zürich); highest level of education (low, middle, high; provided by the Swiss Conference of Cantonal Ministers of Education [19]); source of income (working, retirement pension or social assistance, unemployed); IDU (never, former, current); past and present alcohol consumption (light [<20g/day], moderate [20-40g/day], excessive [>40 g/day], former drinker [reported to have drunk >40 g/day]); time between HCV diagnosis and enrolment into the cohort (<2, 2–5, 6–10, >10 years); calendar year of enrolment (2000–2003, 2004–2007, 2008–2011, 2012–2017); HCV genotype (1, 2, 3, 4; genotypes 5 and 6 were very rare and set to missing); and HIV and/or HBV coinfection. We considered cirrhosis at enrolment (yes/no) as a covariate in all analyses except for cirrhosis at enrolment and incident cirrhosis. The type of treatment—or absence of it—could also influence the outcomes. We therefore adjusted the models on SVR, incident cirrhosis, mortality, LTFU and treatment (not treated, treated with direct-acting antivirals [DAA], treated with non-DAA regimens). Since SVR was only analysed in treated persons, we adjusted this analysis only for type of treatment (DAA versus non-DAA) and considered only treatments before the event of interest.

Our first analysis combined all foreign-born persons into one group. Our second analysis grouped people into the geographic units described above. We performed a main analysis, and two sensitivity analyses where we either imputed or excluded missing values. In the main analysis, we assumed HBV and HIV status were negative if missing. We used multiple imputation by chained equations (MICE package for R [20]) to impute missing values. Because adding variables that are not part of the analysis can improve imputation [21], we added body weight, diabetes comorbidity, and a binary indicator for pre-treatment before cohort enrolment. We also included the outcome of interest. We ran the models on 20 imputed datasets for each analysis and used Rubin’s rule to pool the estimates. All analyses were performed with R version 3.3.3 (R core team, 2016).

## Results

We included 5,356 persons enrolled into the SCCS. The majority were male (62.3%), and the median age at enrolment was 45.3 years (Table 1).

**Table 1:** Baseline characteristics (including test for difference in proportions) of the Swiss Hepatitis C Cohort Study participants, by geographic origin.

		Switzer-land	All foreign born	Germany	Italy	Portugal	Eastern Europe	Southern Europe	Western Europe	Asia and Oceania	Africa	America	N
N		3604 (67.3)	1752 (32.7)	121 (2.3)	546 (10.2)	119 (2.2)	129 (2.4)	202 (3.8)	220 (4.1)	155 (2.9)	160 (3)	100 (1.9)	5356
Gender			P = 0.018	P = 0.13	P = 0.066	P = 0.008	P < 0.001	P = 0.85	P < 0.001	P = 0.72	P = 0.2	P = 0.043	
	Female	1318 (36.6)	700 (40)	53 (43.8)	177 (32.4)	29 (24.4)	90 (69.8)	72 (35.6)	111 (50.5)	54 (34.8)	67 (41.9)	47 (47)	2018 (37.7)
	Male	2286 (63.4)	1052 (60)	68 (56.2)	369 (67.6)	90 (75.6)	39 (30.2)	130 (64.4)	109 (49.5)	101 (65.2)	93 (58.1)	53 (53)	3338 (62.3)
Age (years)			P < 0.001	P = 0.11	P < 0.001	P = 0.002	P = 0.016	P = 0.48	P = 0.007	P = 0.002	P = 0.21	P = 0.77	
	18–40	1347 (37.4)	545 (31.1)	35 (28.9)	96 (17.6)	50 (42)	61 (47.3)	77 (38.1)	64 (29.1)	79 (51)	49 (30.6)	34 (34)	1892 (35.3)
	41–60	1859 (51.6)	870 (49.7)	68 (56.2)	262 (48)	67 (56.3)	50 (38.8)	98 (48.5)	120 (54.5)	60 (38.7)	91 (56.9)	54 (54)	2729 (51)
	≥ 61	391 (10.8)	335 (19.1)	18 (14.9)	187 (34.2)	1 (0.8)	18 (14)	27 (13.4)	36 (16.4)	16 (10.3)	20 (12.5)	12 (12)	726 (13.6)
	Unknown	7 (0.2)	2 (0.1)	0 (0.0)	1 (0.2)	1 (0.8)	0 (0.0)	0 (0.0)	0 (0.0)	0 (0.0)	0 (0.0)	0 (0.0)	9 (0.2)
Education			P < 0.001	P < 0.001	P < 0.001	P < 0.001	P < 0.001	P < 0.001	P = 0.006	P < 0.001	P < 0.001	P < 0.001	
	Low	650 (18)	503 (28.7)	15 (12.4)	188 (34.4)	68 (57.1)	15 (11.6)	60 (29.7)	34 (15.5)	57 (36.8)	38 (23.8)	28 (28)	1153 (21.5)
	Middle	2318 (64.3)	851 (48.6)	62 (51.2)	273 (50.2)	45 (37.8)	49 (38)	115 (56.9)	131 (59.5)	61 (39.4)	76 (47.5)	38 (38)	3169 (59.2)
	High	581 (16.1)	366 (20.9)	42 (34.7)	74 (13.6)	4 (3.4)	62 (48.1)	26 (12.9)	54 (24.5)	32 (20.6)	40 (25)	32 (32)	947 (17.7)
	Unknown	55 (1.5)	32 (1.8)	10 (1.8)	10 (1.8)	2 (1.7)	3 (2.3)	1 (0.5)	1 (0.5)	5 (3.2)	6 (3.8)	2 (2)	87 (1.6)
Employm.			P = 0.002	P = 0.008	P < 0.001	P = 0.027	P < 0.001	P = 0.63	P = 0.13	P = 0.003	P = 0.74	P = 0.043	
	Unempl.	381 (10.6)	145 (8.3)	9 (7.4)	25 (4.6)	11 (9.2)	17 (13.2)	17 (8.4)	15 (6.8)	26 (16.8)	19 (11.9)	6 (6)	526 (9.8)
	Working	2074 (57.5)	1084 (61.9)	87 (71.9)	304 (55.7)	83 (69.7)	93 (72.1)	119 (58.9)	139 (63.2)	97 (62.6)	92 (57.5)	70 (70)	3158 (59)
	Inval.	1136 (31.5)	512 (29.2)	25 (20.7)	213 (39)	25 (21)	19 (14.7)	62 (30.7)	66 (30)	32 (20.6)	46 (28.7)	24 (24)	1648 (30.8)
	Unknown	13 (0.4)	11 (0.6)	0 (0.0)	4 (0.7)	0 (0.0)	0 (0.0)	4 (2)	0 (0.0)	0 (0.0)	3 (1.9)	0 (0.0)	24 (0.4)
(History of) injection drug use			P < 0.001	P < 0.001	P < 0.001	P = 0.26	P < 0.001	P < 0.001	P = 0.012	P < 0.001	P < 0.001	P < 0.001	
	Not user	1254 (34.8)	1098 (62.7)	68 (56.2)	357 (65.4)	49 (41.2)	107 (82.9)	125 (61.9)	98 (44.5)	106 (68.4)	127 (79.4)	61 (61)	2352 (43.9)
	Former	620 (17.2)	214 (12.2)	18 (14.9)	81 (14.8)	21 (17.6)	10 (7.8)	21 (10.4)	29 (13.2)	15 (9.7)	8 (5)	11 (11)	834 (15.6)
	Current	1691 (46.9)	427 (24.4)	35 (28.9)	105 (19.2)	47 (39.5)	12 (9.3)	56 (27.7)	91 (41.4)	31 (20)	22 (13.8)	28 (28)	2118 (39.5)
	Unknown	39 (1.1)	13 (0.7)	0 (0.0)	3 (0.5)	2 (1.7)	0 (0.0)	0 (0.0)	2 (0.9)	3 (1.9)	3 (1.9)	0 (0)	52 (1)
Alcohol cons.			P < 0.001	P = 0.44	P < 0.001	P = 0.091	P < 0.001	P < 0.001	P = 0.43	P < 0.001	P < 0.001	P = 0.29	
	Light	1791 (49.7)	1037 (59.3)	62 (51.2)	333 (61)	49 (41.2)	90 (69.8)	131 (64.9)	103 (46.8)	100 (64.5)	114 (71.2)	55 (55)	2828 (52.8)
	Moderate	618 (17.1)	313 (17.9)	26 (21.5)	98 (17.9)	30 (25.2)	22 (17.1)	30 (14.9)	41 (18.6)	26 (16.8)	20 (12.5)	20 (20)	931 (17.4)
	Excessive	915 (25.4)	309 (17.6)	27 (22.3)	81 (14.8)	32 (26.9)	12 (9.3)	34 (16.8)	64 (29.1)	23 (14.8)	19 (11.9)	17 (17)	1224 (22.9)
	Former	265 (7.4)	87 (5)	6 (5)	32 (5.9)	7 (5.9)	5 (3.9)	7 (3.5)	12 (5.5)	4 (2.6)	7 (4.4)	7 (7)	352 (6.6)
	Unknown	15 (0.4)	6 (0.3)	0 (0.0)	2 (0.4)	1 (0.8)	0 (0.0)	0 (0.0)	0 (0.0)	2 (1.3)	0 (0.0)	1 (1)	21 (0.4)
From diagnosis to enrolm. (years)			P = 0.0014	P = 0.098	P = 0.48	P = 0.062	P = 0.45	P = 0.46	P = 0.14	P < 0.001	P = 0.015	P = 0.33	
	0–2	1303 (36.2)	725 (41.4)	50 (41.3)	203 (37.2)	55 (46.2)	54 (41.9)	82 (40.6)	93 (42.3)	77 (49.7)	71 (44.4)	40 (40)	2028 (37.9)
	2–6	839 (23.3)	386 (22)	21 (17.4)	118 (21.6)	26 (21.8)	33 (25.6)	44 (21.8)	50 (22.7)	28 (18.1)	37 (23.1)	29 (29)	1225 (22.9)
	6–10	613 (17)	284 (16.2)	14 (11.6)	82 (15)	20 (16.8)	18 (14)	35 (17.3)	40 (18.2)	31 (20)	31 (19.4)	13 (13)	897 (16.7)
	> 10	762 (21.1)	315 (18)	32 (26.4)	125 (22.9)	15 (12.6)	23 (17.8)	35 (17.3)	34 (15.5)	16 (10.3)	18 (11.2)	17 (17)	1077 (20.1)
Calendar year of enrolm.			P = 0.054	P < 0.001	P = 0.0035	P = 0.002	P = 0.002	P = 0.34	P = 0.27	P = 0.091	P = 0.39	P = 0.7	
	2000–03	1431 (39.7)	647 (36.9)	39 (32.2)	230 (42.1)	32 (26.9)	40 (31)	74 (36.6)	92 (41.8)	46 (29.7)	53 (33.1)	41 (41)	2078 (38.8)
	2004–07	953 (26.4)	449 (25.6)	34 (28.1)	107 (19.6)	30 (25.2)	25 (19.4)	64 (31.7)	67 (30.5)	50 (32.3)	49 (29.4)	23 (23)	1402 (26.2)
	2008–11	358 (9.9)	204 (11.6)	26 (21.5)	53 (9.7)	22 (18.5)	16 (12.4)	22 (10.9)	19 (8.6)	17 (11)	16 (10)	13 (13)	562 (10.5)
	2012–17	862 (23.9)	452 (25.8)	22 (18.2)	156 (28.6)	35 (29.4)	48 (37.2)	42 (20.8)	42 (19.1)	42 (27.1)	42 (26.2)	23 (23)	1314 (24.5)
HCV genotype			P < 0.001	P = 0.62	P < 0.001	P = 0.086	P < 0.001	P < 0.001	P = 0.15	P = 0.53	P < 0.001	P < 0.001	
	1	1592 (44.2)	849 (48.5)	64 (52.9)	246 (45.1)	61 (51.3)	86 (66.7)	114 (56.5)	97 (44.1)	68 (43.9)	47 (29.4)	66 (66)	2441 (45.6)
	2	229 (6.4)	169 (9.6)	8 (6.6)	87 (15.9)	2 (1.7)	4 (3.1)	15 (7.49	23 (10.5)	11 (7.1)	12 (7.5)	7 (7)	398 (7.4)
	3	985 (27.3)	324 (18.5)	33 (27.3)	87 (15.9)	28 (23.5)	20 (15.5)	30 (14.9)	56 (25.5)	43 (27.7)	16 (10)	11 (11)	1309 (24.4)
	4	311 (8.6)	172 (9.8)	8 (6.6)	38 (7)	13 (10.9)	3 (2.3)	19 (9.4)	21 (9.5)	8 (5.2)	57 (35.6)	5 (5)	483 (9)
	Unknown	487 (13.5)	238 (13.6)	8 (6.6)	88 (16.1)	15 (12.6)	16 (12.4)	24 (11.9)	23 (10.5)	25 (16.1)	28 (17.5)	11 (11)	725 (13.5)
HIV status			P < 0.001	P = 0.57	P = 0.047	P = 0.68	P = 0.023	P = 0.8	P = 0.32	P = 0.34	P = 0.22	P = 0.096	
	Negative	2450 (68)	1167 (66.6)	84 (69.4)	321 (58.8)	90 (75.6)	82 (63.6)	138 (68.4)	160 (72.7)	98 (63.2)	122 (76.2)	72 (72)	3617 (67.5)
	Positive	241 (6.7)	71 (4.1)	6 (5)	19 (3.5)	7 (5.9)	1 (0.8)	12 (5.9)	11 (5)	6 (3.9)	7 (4.4)	2 (2)	312 (5.8)
	Unknown	913 (25.3)	514 (29.3)	31 (25.6)	206 (37.7)	22 (18.5)	46 (35.7)	52 (25.7)	49 (22.3)	51 (32.9)	31 (19.4)	26 (26)	1427 (26.6)
Chronic HBV infection			P = 0.5	P = 0.84	P = 0.99	P = 1	P = 0.75	P = 0.69	P = 0.58	P = 0.026	P = 0.023	P = 1	
	Negative	2772 (76.9)	1324 (75.6)	86 (71.1)	401 (73.4)	93 (78.2)	96 (73.6)	157 (77.8)	173 (78.6)	123 (79.4)	121 (75.6)	75 (75)	4096 (76.5)
	Positive	58 (1.6)	33 (1.9)	1 (0.8)	9 (1.6)	2 (1.7)	1 (0.8)	2 (1)	2 (0.9)	7 (4.5)	7 (4.4)	2 (2)	91 (1.7)
	Unknown	774 (21.5)	395 (22.5)	34 (28.1)	136 (24.9)	24 (20.2)	33 (25.6)	43 (21.3)	45 (20.5)	25 (16.1)	32 (20)	23 (23)	1169 (21.8)
Enrolm. centre			P < 0.001	P < 0.001	P < 0.001	P < 0.001	P = 0.006	P < 0.001	P < 0.001	P < 0.001	P < 0.001	P = 0.0015	
	Basel	234 (6.5)	118 (6.7)	20 (16.5)	37 (6.8)	4 (3.4)	10 (7.8)	13 (6.4)	10 (4.5)	10 (4.5)	7 (4.4)	6 (6)	352 (6.6)
	Bern	752 (20.9)	253 (14.4)	20 (16.5)	64 (11.7)	18 (15.1)	24 (18.6)	30 (14.9)	19 (8.6)	19 (8.6)	26 (16.2)	16 (16)	1005 (18.8)
	Geneva	149 (4.1)	154 (8.8)	2 (1)	36 (6.6)	13 (10.9)	8 (6.2)	26 (12.9)	33 (15)	33 (15)	24 (15)	6 (6)	303 (5.7)
	Lausanne	165 (4.6)	106 (6.1)	3 (2.5)	24 (4.4)	18 (15.1)	5 (3.9)	9 (4.5)	17 (7.7)	17 (7.7)	12 (7.5)	13 (13)	271 (5.1)
	Lugano	387 (10.7)	252 (14.4)	3 (2.5)	187 (34.3)	3 (2.5)	12 (9.3)	12 (5.9)	9 (4.1)	9 (4.1)	11 (6.9)	11 (11)	639 (11.9)
	Neuchâtel	247 (6.9)	159 (9.1)	1 (0.8)	30 (5.5)	28 (23.5)	7 (5.4)	12 (5.9)	36 (16.4)	36 (16.4)	31 (19.4)	7 (7)	406 (7.6)
	St. Gallen	716 (19.9)	225 (12.8)	20 (16.5)	53 (9.7)	12 (10.1)	11 (8.5)	34 (16.8)	52 (23.6)	52 (23.6)	14 (8.8)	9 (9)	941 (17.6)
	Zürich	954 (26.5)	485 (27.7)	52 (43)	115 (21.1)	23 (19.3)	52 (40.3)	66 (32.7)	44 (20)	44 (20)	35 (21.9)	32 (32)	1439 (26.9)
Cirrhotic at enrolm.			P < 0.001	P = 0.2	P < 0.001	P = 1	P = 0.44	P = 0.82	P = 0.58	P = 0.63	P = 0.89	P = 0.62	
	No	3026 (84)	1402 (80)	96 (79.3)	393 (72)	97 (81.5)	113 (87.6)	166 (82.2)	189 (85.9)	126 (81.3)	135 (84.4)	87 (87)	4428 (82.7)
	Yes	547 (15.2)	330 (18.8)	24 (19.8)	149 (27.3)	17 (14.3)	16 (12.4)	32 (15.8)	30 (13.6)	26 (16.8)	23 (14.4)	13 (13)	877 (16.49)
	Unknown	31 (0.9)	20 (1.1)	1 (0.8)	4 (0.7)	5 (4.2)	0 (0.0)	4 (2)	1 (0.5)	3 (1.9)	2 (1.2)	0 (0.0)	51 (1)

Employm. = employment; inval. = invalid pension, social support. Education reflects the highest level of education reached (Swiss Conference of Cantonal Ministers of Education, 2017). Alcohol cons. (= consumption): no or light: ≥ grams of alcohol per day; moderate: >20–40; excessive: >40; former> 40 before enrolment. Southern Europe = Southern Europe except Italy and Portugal, Western Europe = Western and Northern Europe except Germany, America = North and South America combined. P-values (based on Chi-square tests) show difference in proportion between the Swiss group and the group of considered geographic origin.

**Fig 1:**
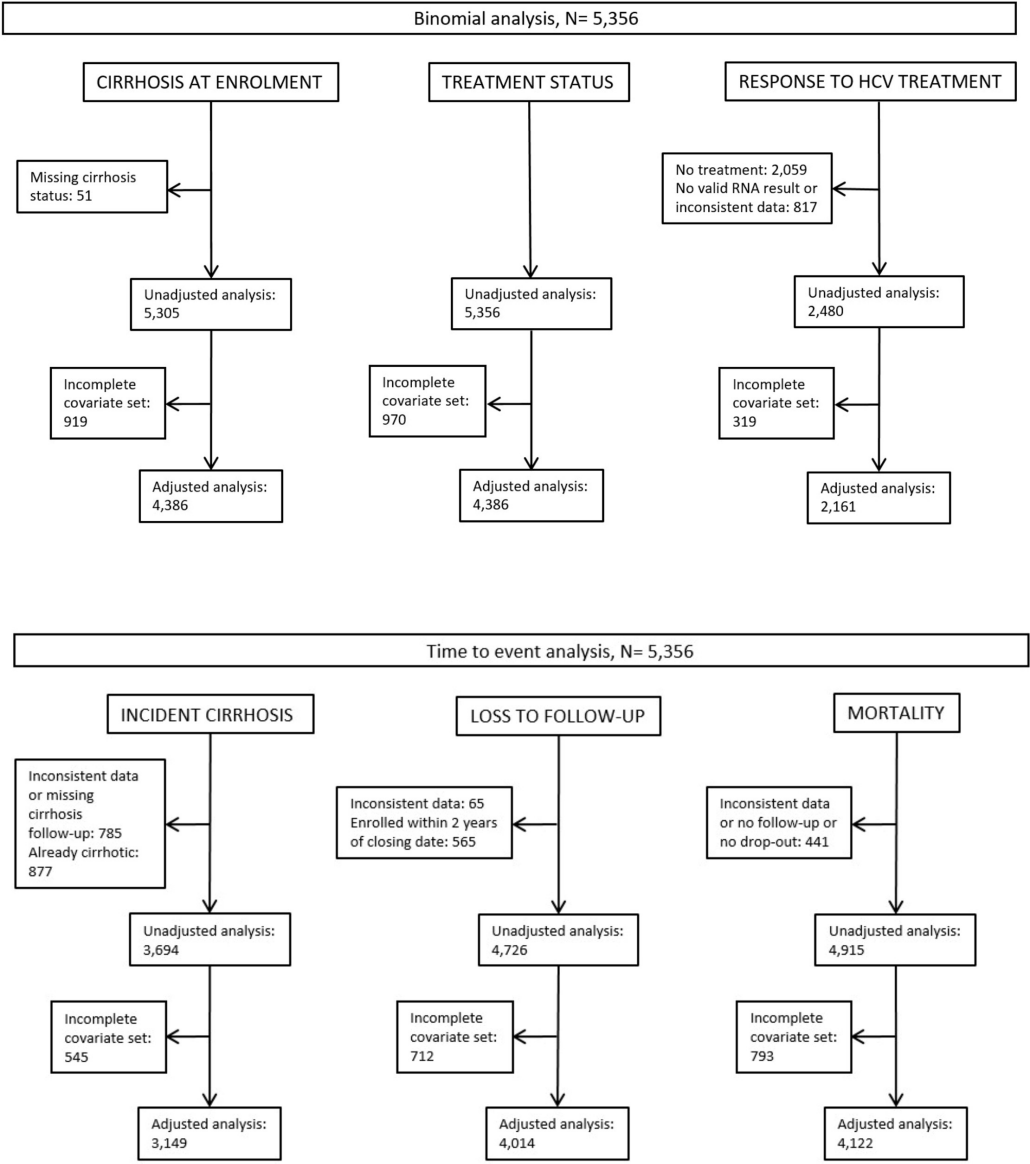
Selection of the Swiss Hepatitis C Cohort Study participants included in the unadjusted and adjusted analyses.

**Fig 2:**
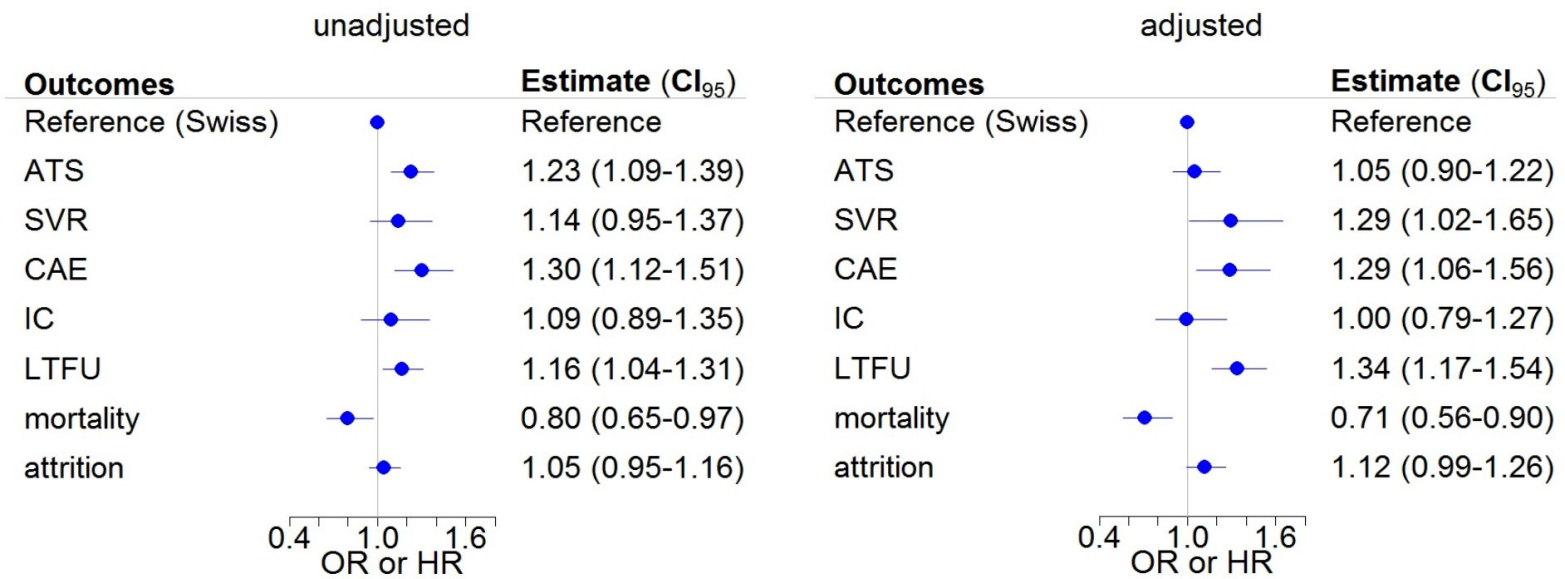
Comparison of Swiss- and foreign-born persons for all analyses. Unadjusted (left) and adjusted (right) logistic and Cox regression analyses for different outcomes of Swiss-born persons (= reference) and foreign-born persons in the Swiss Hepatitis C Cohort Study. Odds ratios (OR; with 95% confidence intervals) are shown for antiviral treatment status (ATS), sustained virologic response (SVR) and cirrhosis at enrolment (CAE). Hazard ratios (HR) are shown for incident cirrhosis (IC), loss to follow-up (LTFU), mortality and attrition.

**Fig 3:**
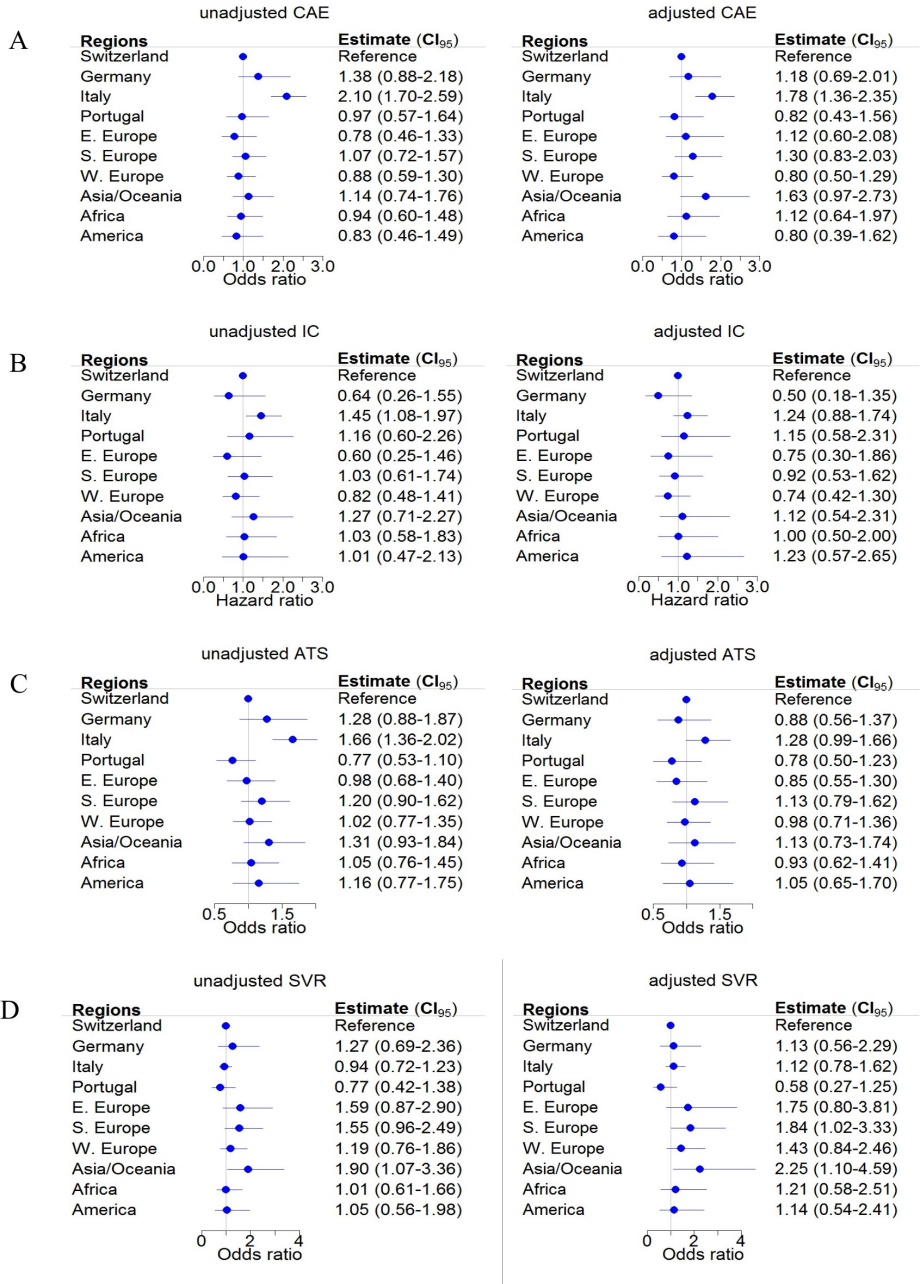
Comparison of persons by geographic origin: cirrhosis and treatment (outcomes). Unadjusted and adjusted logistic and Cox regression analyses by geographic origin of the Swiss Hepatitis C Cohort Study participants. The following outcomes are shown: cirrhosis at enrolment (A), incident cirrhosis (B), antiviral treatment status (C), and sustained virologic response (SVR) (D). Odds ratios, hazard ratios and 95% confidence intervals are presented. Missing HIV and HBV values were assumed to be negative.

**Fig 4:**
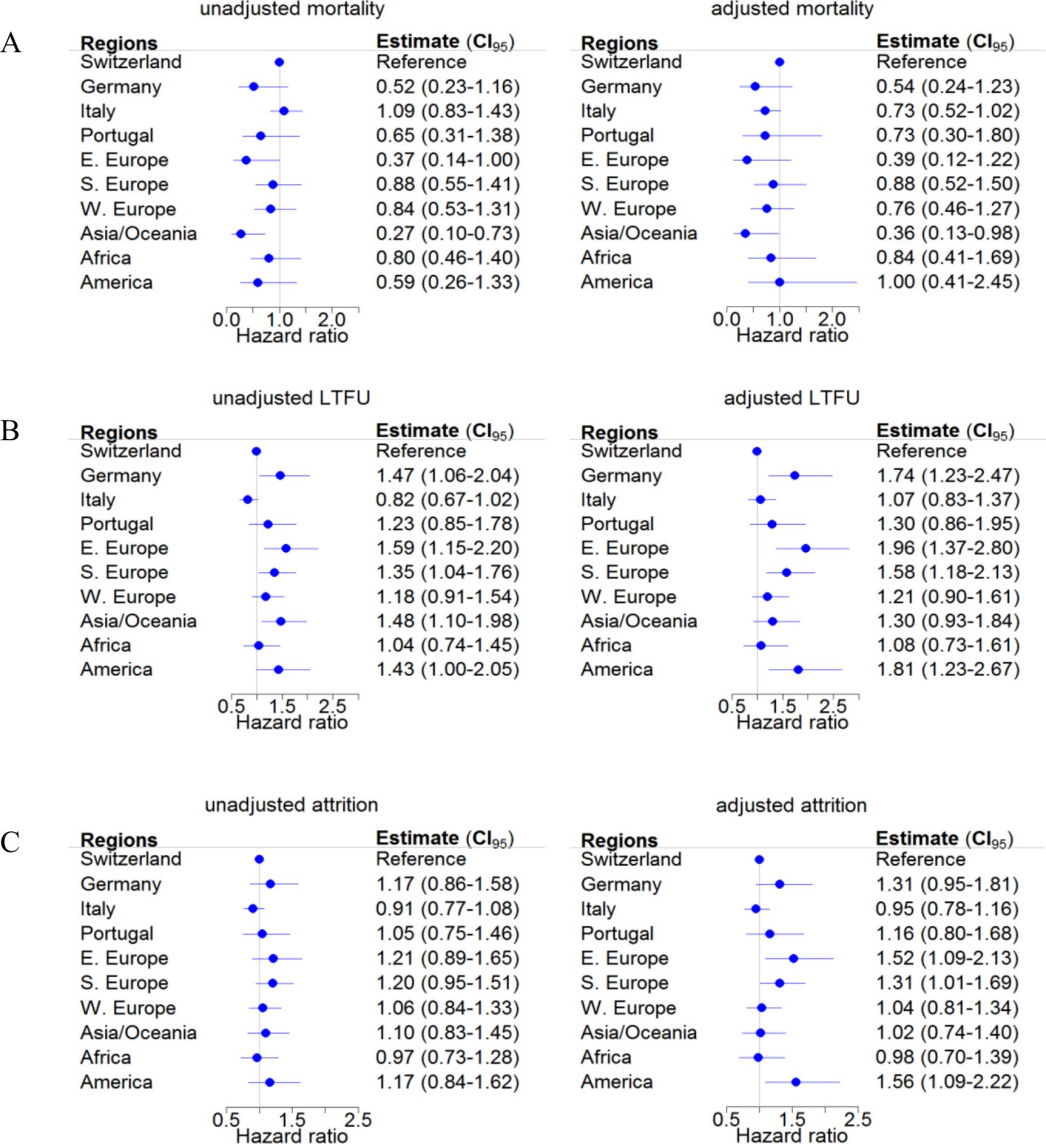
Comparison of persons by geographic origin: mortality, loss to follow-up and attrition. Unadjusted and adjusted Cox regression analyses by geographic origin of the Swiss Hepatitis C Cohort Study participants. The following outcomes are shown: mortality (A), loss to follow-up (B) and attrition (C). Hazard ratios and 95% confidence intervals are presented. Missing HIV and HBV values were assumed to be negative.

Most were current (39.5%) or former (15.6%) IDU; 29.5% reported past or current excessive alcohol use. At enrolment, few had HIV or HBV coinfections (HIV: 67.5% negative and 26.6% missing; HBV: 76.5% negative and 21.8% missing) and most had no cirrhosis (82.7%). Participants were followed for up to 16.6 years (median follow-up duration 7.2 years). Swiss-born participants made up 67.3% of the population, 32.7% were foreign-born (Table 1). Fig 1 shows a flowchart of persons included in the unadjusted and adjusted regression analyses (Fig 1). The number of persons we excluded from the unadjusted analyses varied from 0 (“antiviral treatment status”) to more than 800 people (“response to antivirals”, “incident cirrhosis”). In the adjusted analyses, we excluded between 319–970 persons because of missing values in covariates. S1 Table shows the coefficients for the unadjusted and adjusted models in Figs 2–4. The results of the differences in proportions of each characteristic across the geographic groups are shown in Table 1.

### Comparison of Swiss-born and foreign-born persons

Results of the unadjusted and adjusted analyses are shown in Fig 2. In unadjusted analyses (Fig 2, left panel), foreign-born persons were more likely to have cirrhosis at enrolment (odds ratio [OR], 1.30; 95% confidence interval [CI], 1.12–1.51) and to have ever been on antiviral therapy (OR 1.23; 95% CI, 1.09–1.39) than Swiss-born persons. LTFU was higher (hazard ratio [HR] 1.16; 95% CI 1.04–1.31) and mortality was lower (HR 0.80; 95% CI 0.65–0.97) in foreign- than in Swiss-born persons.

The adjusted analyses confirmed the higher rates of SVR (OR 1.29; 95% CI 1.02–1.65), of cirrhosis at enrollment (OR 1.29; 95% CI 1.06–1.56), and of LTFU (OR 1.34; 95% CI 1.17–1.54) among foreign-born persons (Fig 2, right panel). Mortality was lower among foreign- than Swiss-born persons (OR 0.71; 0.56–0.90). Contrary to the unadjusted analysis, there was no longer a difference in antiviral treatment uptake. When we combined loss to follow-up and mortality into a single attrition outcome, results were similar for foreign- and Swiss- born persons both in the unadjusted and adjusted analyses (Fig 2).

Foreign-born persons LTFU had less often (a history of) IDU than persons not LTFU, while the opposite applied to Swiss-born persons LTFU. Foreign- and Swiss-born persons LTFU were younger and less frequently cirrhotic than persons not LTFU (S2 Table).

### Comparison of different countries and regions

When we compared persons from each region or country to Swiss-born persons, some notable differences emerged. Italian-born participants were more likely to have cirrhosis at enrolment in both the unadjusted and adjusted analyses (Fig 3, panels A), they had more incident cirrhosis during follow-up in the unadjusted analysis (Fig 3, panels B), and they were more likely to be treated than Swiss-born participants in the unadjusted, but not in the adjusted analysis (adjusted OR 1.28; 95% CI 0.99–1.66) (Fig 3, panels C). In the adjusted analysis, persons from Asia/Oceania and Southern Europe were more likely to have SVR than Swiss-born participants (Fig 3, panels D).

In both the unadjusted and adjusted analyses, mortality was lower in those from Asia/Oceania than in people of Swiss origin (Fig 4, panels A). LTFU was higher in both the unadjusted and adjusted analyses in people from Germany (HR 1.74; 95% CI 1.23–2.47), Eastern Europe (HR 1.96; 95% CI 1.37–2.8), Southern Europe (HR 1.58; 95% CI 1.18–2.13) and the Americas (HR 1.81; 95% CI 1.23–2.67) (all data are from the adjusted analysis, Fig 4, panels B, right). Attrition was similar between Swiss- and foreign-born persons from all countries or regions in the unadjusted analysis and, in the adjusted analysis, higher for those from Eastern and Southern Europe and the Americas (Fig 4, panels C).

Results from the sensitivity analyses, where we either replaced missing values with multiple imputations or removed persons with missing values, were similar to the main analyses (S3 Table).

## Discussion

We found that geographic origin did not influence access to antiviral treatment for HCV-infected patients enrolled in the SCCS. We did find a few notable differences between foreign-born and Swiss-born persons: although cirrhosis at enrolment was higher among foreign- than Swiss-born persons, the higher SVR probably lowered mortality among foreign-born persons. LTFU varied substantially between groups of different origin.

### Cirrhosis at enrolment

The higher prevalence of cirrhosis at enrolment among foreign-born participants was the result of the high prevalence of cirrhosis at enrolment among persons from Italy, the largest group of foreign-born people in Switzerland (Swiss Federal Statistical Office 2017) and in the SCCS (Table 1). Many Italian-born persons had acquired HCV in Italian healthcare settings between 1950 and 1970 [12,13]. These people were often diagnosed long after they were infected with HCV [15] and tended to be older than Swiss-born people with HCV, which increased their risk of presenting with cirrhosis [15].

### Antiviral treatment status, SVR and incident cirrhosis

The fact that there are no differences in antiviral treatment status and incident cirrhosis between Swiss- born and foreign-born persons is reassuring. Even though foreign-born persons may have faced obstacles including language problems or cultural differences, these did not negatively affect the uptake of HCV therapy. In the comparison of different countries and regions, antiviral treatment coverage was higher in Italian-born persons than in all other groups including Swiss-born persons, but that difference disappeared in the adjusted analysis. Many Italian-born residents of Switzerland live in the Italian-speaking canton of Ticino close to the Italian border. In Ticino, efforts to find HCV-infected Italian-born persons have been ongoing for many years, with the involvement of general practitioners. HCV treatment in Ticino is decentralized into smaller facilities, which may have facilitated earlier access to therapy and improved therapy coverage. The high prevalence of cirrhosis at enrolment in this population further encouraged early treatment, including treatment with DAA.

The higher SVR among foreign-born persons in the adjusted analysis, despite their higher proportion of cirrhosis, may be a consequence of the lower prevalence of IDU and of less alcohol consumption among foreign-born persons (Table 1). Although Switzerland offers a wide range of treatment options for people with (a history of) IDU [22], there are still some gaps in the treatment cascade [22–24], and these may have lowered the rate of treatment and of SVR among persons with (a history of) IDU in Switzerland.

To our knowledge, there are no previous publications from Europe on the impact of geographic origin on access to treatment and treatment outcomes in persons born in the respective country compared to foreign-born persons. In the US, having a health insurance was associated with improved access to HCV care [25,26], and disparities in access to health insurance have been observed among ethnic minority groups [26]. In contrast, our data from a country with equal access to healthcare for all residents show that similar outcomes can be achieved, irrespective of country of origin. This is relevant, as the proportion of HCV-infected foreign-born persons among all HCV infections is up to one third in European countries and in the US [27,28], and may be similarly high in other resource-rich countries. These good outcomes for foreign-born persons with HCV infection should be achievable not only in countries with universal healthcare and predominantly European migrants (e.g. Switzerland) [15], but also in countries with universal healthcare and many non-European migrants (e.g. the Netherlands and the UK) [15]. This would follow the example of Canada, where similar access to treatment and similar treatment outcomes were recently shown for Canadian- and foreign-born persons from highly diverse countries of origin [29].

### Loss to follow-up

Higher LTFU among foreign-born persons was driven by immigrants from Eastern and Southern Europe, the Americas, and Germany and probably reflects migration patterns in Switzerland, since many persons from these countries come to Switzerland for employment and then return to their home countries [30]. These persons are recommended to continue HCV care in their home countries. Differences in LTFU may also be caused by center-specific factors, including variable resources for tracing patients.

Among foreign-born persons LTFU, there may be persons who emigrated and died in their country of destination. However, numbers are likely low, as only 39 foreign-born persons LTFU were older than 60 years, and foreign-born persons LTFU were less frequently cirrhotic at enrolment than persons not LTFU (S2 Table).

### Mortality

The higher prevalence of cirrhosis at enrolment among many Italian-born residents did not increase mortality (Fig 4).This is likely the result of their good treatment coverage, although the better coverage in the unadjusted analysis disappeared in the adjusted analysis (Fig 3). Although Swiss-born persons were generally younger than foreign-born persons, they did not live longer, perhaps because they were more likely to have a history of IDU. For IDU, the probability of being treated is often influenced by psychosocial and socio-economic problems [31,32], and by increased alcohol consumption [33]. Although our multivariable models accounted for IDU, we could not adjust for intensity of drug use, and we had no information on other associated lifestyle factors like dietary habits, physical activity, and smoking behaviour. Only 5.8% of the persons in our study population were HIV-positive, but HIV infection, a risk factor for accelerated HCV disease progression, was also more frequent among Swiss-born persons (6.7% vs. 4.1%).

It is difficult to estimate the contribution of the “healthy migrant effect”. This effect has been shown in the European Union (EU), where out of 10 anti-HCV prevalence estimates among the general migrant population, 70% were comparable to the in-country prevalence and 30% were lower [34]. In Switzerland, this effect may be counterbalanced by the high number of migrants from Italy, the EU country with the highest prevalence of HCV infections [35].

### Strengths and limitations

Our study was strengthened by the large number of persons enrolled into the cohort, and the long follow-up period. Agreement between complete case analysis results and imputed analysis results supports the validity of our findings. Most differences in outcomes were caused by variation in the size of the confidence intervals and reflect differences in sample size rather than effect size.

Our study may be limited in its generalizability for three reasons. First, Switzerland has a predominantly European migration pattern [9]. Second, most groups of foreign-born people were small, which limited the power of our analysis. To reach a reasonable sample size we had to group people from culturally diverse regions (e.g., “Asia/Oceania” includes people from Eastern Asian and Arab countries). Third, SCCS participants are likely to be in a more advanced stage of the disease than HCV-infected persons in the general Swiss population [17].

## Conclusions

We found that in Switzerland, a country with universal healthcare, geographic origin had no influence on access to hepatitis C treatment. Foreign-born persons had more frequent SVR and lower mortality despite more frequent cirrhosis at enrolment, and they were more often LTFU. The high frequency of cirrhosis at enrolment among foreign-borns mirrors the high rate of cirrhosis at enrolment among Italian-born persons who have acquired HCV between 1950 and 1970 in Italian healthcare settings. However since most of the Italian-born persons were treated, mortality was not increased. The better treatment outcomes among foreign-born persons were likely explained by their lower prevalence of IDU and of alcohol consumption than among Swiss-born persons.

## Supporting information

S1 Table All odds ratios / hazard ratios included in the models. (PDF)

S2 Table Characteristics of Swiss-/foreign-born persons LTFU compared to persons not LTFU. (PDF)

S3 Table Sensitivity analysis: Comparison of Swiss-born and foreign-born persons, with calculation of missing values by multiple imputation. (PDF)
