# Supplementary material for: Impact of geographic origin on access to therapy and therapy outcomes in the Swiss Hepatitis C Cohort Study
Source: PLoS One. 2019 Jun 24;14(6):e0218706. doi: 10.1371/journal.pone.0218706 (PMC6590815; doi:10.1371/journal.pone.0218706)
Supplement: S1 Table — (PDF) [file pone.0218706.s001.pdf]

S1 Table: All odds ratios / hazard ratios included in the models

For the comparison of Swiss-born and foreign-born persons (adjusted models)

		ATS	SVR	CAE	IC	LTFU	mortality	attrition
Swiss-/Foreign-born		P = 0.66	P = 0.039	P = 0.0099	P = 1	P < 0.001	P = 0.0032	P = 0.04
	Swiss-born	1.0 (ref.)	1.0 (ref.)	1.0 (ref.)	1.0 (ref.)	1.0 (ref.)	1.0 (ref.)	1.0 (ref.)
	Foreign-born	1.05 (0.9-1.22)	1.29 (1.02-1.65)	1.29 (1.06-1.56)	1 (0.79-1.27)	1.34 (1.17-1.54)	0.71 (0.56-0.9)	1.12 (0.99-1.26)
Gender		P < 0.001	P = 0.1	P < 0.001	P = 0.0029	P = 0.79	P < 0.001	P = 0.0034
	Female	1.0 (ref.)	1.0 (ref.)	1.0 (ref.)	1.0 (ref.)	1.0 (ref.)	1.0 (ref.)	1.0 (ref.)
	Male	1.46 (1.27-1.68)	0.87 (0.69-1.1)	1.7 (1.4-2.07)	1.41 (1.12-1.79)	0.99 (0.87-1.13)	1.36 (1.09-1.71)	1.08 (0.97-1.21)
Age (y)		P < 0.001	P = 0.041	P < 0.001	P < 0.001	P < 0.001	P < 0.001	P < 0.001
	18-40	1.0 (ref.)	1.0 (ref.)	1.0 (ref.)	1.0 (ref.)	1.0 (ref.)	1.0 (ref.)	1.0 (ref.)
	41-60	1.59 (1.37-1.85)	0.65 (0.51-0.83)	4.89 (3.76-6.36)	2.67 (2.03-3.5)	0.73 (0.64-0.84)	1.67 (1.29-2.15)	0.86 (0.76-0.96)
	≥ 61	0.95 (0.72-1.24)	0.53 (0.34-0.83)	8.7 (6.05-12.49)	4.48 (2.94-6.84)	0.79 (0.59-1.05)	3.29 (2.23-4.85)	1.21 (0.97-1.51)
Education		P = 0.31	P = 0.014	P = 0.0054	P = 0.03	P = 0.1	P = 0.091	P = 0.0039
	Low	0.91 (0.77-1.08)	0.97 (0.73-1.29)	1.18 (0.94-1.47)	1.34 (1.03-1.74)	1.11 (0.95-1.29)	1.11 (0.87-1.41)	1.09 (0.96-1.24)
	Middle	1.0 (ref.)	1.0 (ref.)	1.0 (ref.)	1.0 (ref.)	1.0 (ref.)	1.0 (ref.)	1.0 (ref.)
	High	0.86 (0.71-1.03)	1.46 (1.08-1.97)	0.9 (0.71-1.14)	1.01 (0.76-1.36)	1.03 (0.86-1.23)	1.05 (0.79-1.39)	1.05 (0.9-1.22)
Employment		P < 0.001	P = 0.23	P < 0.001	P = 0.1	P < 0.001	P < 0.001	P < 0.001
	Unempl.	1.0 (ref.)	1.0 (ref.)	1.0 (ref.)	1.0 (ref.)	1.0 (ref.)	1.0 (ref.)	1.0 (ref.)
	Working	1.27 (1-1.6)	0.75 (0.5-1.11)	1.11 (0.77-1.59)	0.93 (0.62-1.39)	0.71 (0.59-0.85)	0.64 (0.46-0.9)	0.68 (0.58-0.8)
	Inval.	1.03 (0.8-1.33)	0.53 (0.34-0.83)	1.59 (1.09-2.33)	1.07 (0.69-1.64)	0.69 (0.56-0.86)	1.16 (0.82-1.65)	0.82 (0.69-0.98)
(History of) injection drug use		P < 0.001	P < 0.001	P = 0.052	P = 0.043	P = 0.021	P = 0.087	P = 0.019
	Not user	1.0 (ref.)	1.0 (ref.)	1.0 (ref.)	1.0 (ref.)	1.0 (ref.)	1.0 (ref.)	1.0 (ref.)
	Former	0.65 (0.51-0.83)	1.05 (0.66-1.69)	0.59 (0.43-0.81)	1.04 (0.69-1.56)	1.14 (0.87-1.49)	1.31 (0.84-2.03)	1.13 (0.9-1.43)
	Current	0.66 (0.55-0.79)	1.07 (0.81-1.4)	0.55 (0.43-0.69)	0.76 (0.57-1.01)	1.35 (1.15-1.59)	0.87 (0.66-1.13)	1.17 (1.02-1.35)
Alcohol consumption		P = 0.022	P = 0.11	P < 0.001	P = 0.0059	P = 0.11	P < 0.001	P < 0.001
	Light	1.0 (ref.)	1.0 (ref.)	1.0 (ref.)	1.0 (ref.)	1.0 (ref.)	1.0 (ref.)	1.0 (ref.)
	Moderate	1.17 (0.96-1.41)	1.1 (0.81-1.49)	1.22 (0.94-1.57)	1.13 (0.83-1.53)	0.88 (0.74-1.06)	1.15 (0.85-1.55)	0.92 (0.78-1.07)
	Excessive	0.86 (0.72-1.03)	0.77 (0.58-1.04)	2.99 (2.37-3.76)	1.61 (1.21-2.14)	1.01 (0.86-1.18)	2.06 (1.6-2.65)	1.2 (1.05-1.37)
	Former	1.08 (0.81-1.44)	0.9 (0.55-1.45)	2.51 (1.78-3.55)	1.57 (1.03-2.4)	0.79 (0.6-1.05)	1.66 (1.15-2.4)	0.96 (0.78-1.2)
Time from diagnosis to enrolment (y)		P < 0.001	P < 0.001	P = 0.048	P = 0.32	P < 0.001	P = 0.32	P < 0.001
	0-2	1.0 (ref.)	1.0 (ref.)	1.0 (ref.)	1.0 (ref.)	1.0 (ref.)	1.0 (ref.)	1.0 (ref.)
	2-6	1.35 (1.14-1.61)	0.59 (0.44-0.78)	1.07 (0.85-1.36)	1.14 (0.86-1.5)	0.82 (0.7-0.96)	0.81 (0.63-1.05)	0.82 (0.72-0.93)
	6-10	1.64 (1.34-1.99)	0.66 (0.49-0.9)	1.19 (0.92-1.54)	1.2 (0.89-1.62)	0.79 (0.66-0.94)	1.04 (0.81-1.35)	0.84 (0.73-0.98)
	10+	1.56 (1.28-1.9)	0.53 (0.39-0.72)	1.33 (1.04-1.69)	1.29 (0.95-1.75)	0.66 (0.54-0.82)	0.9 (0.67-1.2)	0.72 (0.61-0.85)
Calendar year of enrolment		P < 0.001	P = 0.3	P = 0.85	P = 0.056	P < 0.001	P = 0.65	P < 0.001
	2000-2003	1.0 (ref.)	1.0 (ref.)	1.0 (ref.)	1.0 (ref.)	1.0 (ref.)	1.0 (ref.)	1.0 (ref.)
	2004-2007	0.72 (0.61-0.85)	0.95 (0.74-1.22)	1.02 (0.82-1.28)	1.27 (0.99-1.62)	1.59 (1.38-1.82)	1.12 (0.9-1.39)	1.45 (1.29-1.62)
	2008-2011	0.77 (0.6-0.99)	0.96 (0.62-1.48)	1.09 (0.8-1.49)	1.17 (0.76-1.8)	2.53 (2.05-3.13)	0.9 (0.59-1.38)	2.01 (1.67-2.43)
	2012-2017	0.51 (0.41-0.64)	0.59 (0.34-1.02)	0.94 (0.71-1.26)	1.82 (1.13-2.92)	1.95 (1.37-2.78)	0.97 (0.51-1.87)	1.66 (1.22-2.27)
HCV genotype		P < 0.001	P < 0.001	P < 0.001	P = 0.0035	P < 0.001	P = 0.02	P < 0.001
	1	1.0 (ref.)	1.0 (ref.)	1.0 (ref.)	1.0 (ref.)	1.0 (ref.)	1.0 (ref.)	1.0 (ref.)
	2	1.11 (0.86-1.43)	3.68 (2.48-5.48)	0.51 (0.36-0.72)	0.7 (0.48-1.04)	1.02 (0.78-1.33)	0.74 (0.51-1.09)	0.89 (0.72-1.11)
	3	1.44 (1.23-1.69)	2.6 (2.02-3.35)	1.57 (1.27-1.94)	1.43 (1.11-1.84)	1.37 (1.19-1.58)	0.93 (0.74-1.17)	1.24 (1.1-1.4)
	4	0.67 (0.54-0.83)	1.4 (0.91-2.14)	0.98 (0.72-1.34)	0.84 (0.56-1.25)	1.22 (0.99-1.49)	0.75 (0.52-1.07)	1.08 (0.9-1.28)
HIV status		P < 0.001	P = 0.43	P = 0.49	P = 0.21	P < 0.001	P < 0.001	P = 0.6
	Negative	1.0 (ref.)	1.0 (ref.)	1.0 (ref.)	1.0 (ref.)	1.0 (ref.)	1.0 (ref.)	1.0 (ref.)
	Positive	0.33 (0.24-0.44)	0.68 (0.38-1.23)	1.25 (0.83-1.87)	1.22 (0.79-1.88)	0.58 (0.43-0.8)	1.92 (1.4-2.64)	0.94 (0.76-1.17)
Chronic HBV infection		P = 0.72	P = 0.85	P = 0.092	P = 0.55	P = 0.016	P = 0.37	P = 0.17
	Negative	1.0 (ref.)	1.0 (ref.)	1.0 (ref.)	1.0 (ref.)	1.0 (ref.)	1.0 (ref.)	1.0 (ref.)
	Positive	0.99 (0.57-1.73)	0.96 (0.4-2.26)	1.73 (0.94-3.19)	1.26 (0.59-2.68)	0.5 (0.25-1.01)	1.43 (0.78-2.64)	0.8 (0.51-1.26)
Enrolment centre		P < 0.001	P < 0.001	P < 0.001	P = 0.2	P < 0.001	P = 0.03	P < 0.001
	Basel	1.0 (ref.)	1.0 (ref.)	1.0 (ref.)	1.0 (ref.)	1.0 (ref.)	1.0 (ref.)	1.0 (ref.)
	Bern	0.14 (0.1-0.2)	0.35 (0.23-0.53)	0.77 (0.53-1.11)	1.13 (0.68-1.89)	1.46 (1.1-1.94)	1.39 (0.92-2.11)	1.46 (1.16-1.85)
	Geneva	0.33 (0.22-0.51)	0.54 (0.33-0.89)	0.59 (0.37-0.94)	1.54 (0.88-2.7)	1.06 (0.74-1.53)	1.28 (0.77-2.1)	1.14 (0.85-1.54)
	Lausanne	0.24 (0.16-0.37)	0.43 (0.24-0.76)	1.05 (0.66-1.66)	1.25 (0.67-2.32)	0.9 (0.6-1.34)	0.71 (0.4-1.27)	0.85 (0.61-1.19)
	Lugano	0.34 (0.23-0.51)	0.59 (0.34-1.01)	0.6 (0.39-0.92)	1.26 (0.72-2.21)	1.07 (0.74-1.56)	1.15 (0.66-2)	1.06 (0.78-1.44)
	Neuch?tel	0.29 (0.2-0.44)	0.93 (0.55-1.56)	0.41 (0.26-0.66)	0.84 (0.45-1.56)	1.47 (1.06-2.02)	1.29 (0.77-2.19)	1.41 (1.07-1.85)
	St-Gall	0.37 (0.26-0.53)	1.15 (0.72-1.83)	0.43 (0.29-0.64)	1.42 (0.87-2.31)	1.55 (1.17-2.06)	1.42 (0.92-2.19)	1.5 (1.18-1.9)
	Z?rich	0.43 (0.31-0.62)	0.63 (0.43-0.93)	0.75 (0.53-1.07)	1.01 (0.62-1.65)	1.64 (1.26-2.14)	1.26 (0.84-1.89)	1.53 (1.22-1.9)
Cirrhotic at enrolment		P < 0.001	P = 0.021	X	X	P < 0.001	P < 0.001	P < 0.001
	No	1.0 (ref.)	1.0 (ref.)	X	X	1.0 (ref.)	1.0 (ref.)	1.0 (ref.)
	Yes	3 (2.4-3.76)	0.67 (0.5-0.88)	X	X	0.69 (0.55-0.87)	4.54 (3.66-5.62)	1.62 (1.4-1.86)
Treatment		X	X	X	P = 0.32	P < 0.001	P < 0.001	P < 0.001
	No treatment	X	X	X	1.0 (ref.)	1.0 (ref.)	1.0 (ref.)	1.0 (ref.)
	Treated with DAA	X	X	X	0.93 (0.64-1.36)	0.07 (0.04-0.14)	0.16 (0.09-0.28)	0.11 (0.07-0.16)
	Treated without DAA	X	X	X	0.9 (0.71-1.13)	1.1 (0.96-1.25)	0.74 (0.6-0.91)	0.98 (0.88-1.1)
Ever received DAA		X	P < 0.001	X	X	X	X	X
	No	X	1.0 (ref.)	X	X	X	X	X
	Yes	X	33.55 (20.49-54.94)	X	X	X	X	X

OR= odds ratio, with 95% confidence interval; HR= hazard ratio with 95% confidence interval.
ATS= antiviral treatment status; SVR= sustained virologic response; CAE= cirrhosis at enrolment; IC= incident cirrhosis during follow-up; LTFU= loss to follow-up.
DAA= direct-acting antivirals.

For the comparison of persons by geographic origin (adjusted models)

		ATS	SVR	CAE	IC	LTFU	mortality	attrition
Geographic origin		P = 0.37	P = 0.11	P = 0.0061	P = 0.7	P < 0.001	P = 0.11	P = 0.018
	Switzerland	1.0 (ref.)	1.0 (ref.)	1.0 (ref.)	1.0 (ref.)	1.0 (ref.)	1.0 (ref.)	1.0 (ref.)
	Germany	0.88 (0.56-1.37)	1.13 (0.56-2.29)	1.18 (0.69-2.01)	0.5 (0.18-1.35)	1.74 (1.23-2.47)	0.54 (0.24-1.23)	1.31 (0.95-1.81)
	Italy	1.28 (0.99-1.66)	1.12 (0.78-1.62)	1.78 (1.36-2.35)	1.24 (0.88-1.74)	1.07 (0.83-1.37)	0.73 (0.52-1.02)	0.95 (0.78-1.16)
	Portugal	0.78 (0.5-1.23)	0.58 (0.27-1.25)	0.82 (0.43-1.56)	1.15 (0.58-2.31)	1.3 (0.86-1.95)	0.73 (0.3-1.8)	1.16 (0.8-1.68)
	Eastern Europe	0.85 (0.55-1.3)	1.75 (0.8-3.81)	1.12 (0.6-2.08)	0.75 (0.3-1.86)	1.96 (1.37-2.8)	0.39 (0.12-1.22)	1.52 (1.09-2.13)
	Southern Europe	1.13 (0.79-1.62)	1.84 (1.02-3.33)	1.3 (0.83-2.03)	0.92 (0.53-1.62)	1.58 (1.18-2.13)	0.88 (0.52-1.5)	1.31 (1.01-1.69)
	Western Europe	0.98 (0.71-1.36)	1.43 (0.84-2.46)	0.8 (0.5-1.29)	0.74 (0.42-1.3)	1.21 (0.9-1.61)	0.76 (0.46-1.27)	1.04 (0.81-1.34)
	Asia/Oceania	1.13 (0.73-1.74)	2.25 (1.1-4.59)	1.63 (0.97-2.73)	1.12 (0.54-2.31)	1.3 (0.93-1.84)	0.36 (0.13-0.98)	1.02 (0.74-1.4)
	Africa	0.93 (0.62-1.41)	1.21 (0.58-2.51)	1.12 (0.64-1.97)	1 (0.5-2)	1.08 (0.73-1.61)	0.84 (0.41-1.69)	0.98 (0.7-1.39)
	America	1.05 (0.65-1.7)	1.14 (0.54-2.41)	0.8 (0.39-1.62)	1.23 (0.57-2.65)	1.81 (1.23-2.67)	1 (0.41-2.45)	1.56 (1.09-2.22)
Gender		P < 0.001	P = 0.1	P < 0.001	P = 0.0029	P = 0.79	P < 0.001	P = 0.0034
	Female	1.0 (ref.)	1.0 (ref.)	1.0 (ref.)	1.0 (ref.)	1.0 (ref.)	1.0 (ref.)	1.0 (ref.)
	Male	1.44 (1.25-1.66)	0.89 (0.7-1.13)	1.63 (1.33-1.99)	1.36 (1.07-1.73)	1.01 (0.89-1.16)	1.36 (1.08-1.7)	1.1 (0.98-1.23)
Age (y)		P < 0.001	P = 0.041	P < 0.001	P < 0.001	P < 0.001	P < 0.001	P < 0.001
	18-40	1.0 (ref.)	1.0 (ref.)	1.0 (ref.)	1.0 (ref.)	1.0 (ref.)	1.0 (ref.)	1.0 (ref.)
	41-60	1.58 (1.35-1.84)	0.66 (0.52-0.85)	4.86 (3.73-6.33)	2.66 (2.03-3.5)	0.75 (0.65-0.86)	1.66 (1.29-2.14)	0.87 (0.77-0.97)
	≥ 61	0.91 (0.69-1.2)	0.56 (0.36-0.88)	8.28 (5.73-11.96)	4.4 (2.86-6.77)	0.83 (0.62-1.12)	3.25 (2.19-4.81)	1.25 (1-1.56)
Education		P = 0.31	P = 0.014	P = 0.0054	P = 0.03	P = 0.1	P = 0.091	P = 0.0039
	Low	0.91 (0.76-1.08)	1.01 (0.76-1.35)	1.14 (0.91-1.43)	1.28 (0.98-1.67)	1.12 (0.96-1.3)	1.11 (0.87-1.42)	1.1 (0.96-1.25)
	Middle	1.0 (ref.)	1.0 (ref.)	1.0 (ref.)	1.0 (ref.)	1.0 (ref.)	1.0 (ref.)	1.0 (ref.)
	High	0.87 (0.73-1.05)	1.42 (1.05-1.93)	0.94 (0.74-1.2)	1.05 (0.78-1.4)	1 (0.83-1.2)	1.06 (0.8-1.41)	1.03 (0.88-1.19)
Employment		P < 0.001	P = 0.23	P < 0.001	P = 0.1	P < 0.001	P < 0.001	P < 0.001
	Unempl.	1.0 (ref.)	1.0 (ref.)	1.0 (ref.)	1.0 (ref.)	1.0 (ref.)	1.0 (ref.)	1.0 (ref.)
	Working	1.26 (1-1.6)	0.77 (0.51-1.14)	1.1 (0.76-1.58)	0.91 (0.61-1.37)	0.71 (0.59-0.85)	0.64 (0.45-0.9)	0.68 (0.58-0.8)
	Inval.	1.02 (0.79-1.32)	0.53 (0.34-0.83)	1.57 (1.07-2.29)	1.05 (0.68-1.62)	0.7 (0.56-0.86)	1.16 (0.82-1.65)	0.82 (0.69-0.98)
(History of) injection drug use		P < 0.001	P < 0.001	P = 0.052	P = 0.043	P = 0.021	P = 0.087	P = 0.019
	Not user	1.0 (ref.)	1.0 (ref.)	1.0 (ref.)	1.0 (ref.)	1.0 (ref.)	1.0 (ref.)	1.0 (ref.)
	Former	0.65 (0.51-0.82)	1.08 (0.67-1.74)	0.59 (0.43-0.81)	1.03 (0.69-1.56)	1.16 (0.88-1.53)	1.32 (0.85-2.05)	1.15 (0.91-1.45)
	Current	0.66 (0.55-0.79)	1.09 (0.83-1.43)	0.55 (0.43-0.7)	0.77 (0.58-1.02)	1.38 (1.17-1.62)	0.87 (0.66-1.13)	1.18 (1.03-1.36)
Alcohol consumption		P = 0.022	P = 0.11	P < 0.001	P = 0.0059	P = 0.11	P < 0.001	P < 0.001
	Light	1.0 (ref.)	1.0 (ref.)	1.0 (ref.)	1.0 (ref.)	1.0 (ref.)	1.0 (ref.)	1.0 (ref.)
	Moderate	1.18 (0.97-1.43)	1.12 (0.82-1.52)	1.25 (0.97-1.62)	1.13 (0.83-1.54)	0.88 (0.74-1.06)	1.14 (0.84-1.55)	0.92 (0.78-1.07)
	Excessive	0.87 (0.73-1.04)	0.79 (0.59-1.06)	3.11 (2.47-3.93)	1.65 (1.24-2.21)	1 (0.85-1.17)	2.04 (1.59-2.63)	1.19 (1.04-1.36)
	Former	1.09 (0.82-1.45)	0.93 (0.57-1.51)	2.58 (1.82-3.65)	1.6 (1.05-2.45)	0.79 (0.6-1.03)	1.67 (1.15-2.41)	0.96 (0.77-1.19)
Time from diagnosis to enrolment (y)		P < 0.001	P < 0.001	P = 0.048	P = 0.32	P < 0.001	P = 0.32	P < 0.001
	0-2	1.0 (ref.)	1.0 (ref.)	1.0 (ref.)	1.0 (ref.)	1.0 (ref.)	1.0 (ref.)	1.0 (ref.)
	2-6	1.35 (1.14-1.61)	0.58 (0.44-0.78)	1.08 (0.85-1.37)	1.15 (0.87-1.52)	0.82 (0.7-0.96)	0.81 (0.63-1.04)	0.82 (0.72-0.93)
	6-10	1.64 (1.34-1.99)	0.66 (0.49-0.89)	1.2 (0.93-1.55)	1.22 (0.9-1.65)	0.79 (0.66-0.94)	1.04 (0.8-1.35)	0.84 (0.73-0.98)
	10+	1.55 (1.27-1.89)	0.53 (0.39-0.72)	1.31 (1.03-1.67)	1.29 (0.95-1.76)	0.66 (0.53-0.82)	0.89 (0.67-1.19)	0.72 (0.61-0.85)
Calendar year of enrolment		P < 0.001	P = 0.28	P = 0.88	P = 0.057	P < 0.001	P = 0.64	P < 0.001
	2000-2003	1.0 (ref.)	1.0 (ref.)	1.0 (ref.)	1.0 (ref.)	1.0 (ref.)	1.0 (ref.)	1.0 (ref.)
	2004-2007	0.73 (0.62-0.86)	0.95 (0.74-1.22)	1.04 (0.83-1.3)	1.27 (0.99-1.62)	1.58 (1.38-1.81)	1.12 (0.9-1.39)	1.45 (1.29-1.62)
	2008-2011	0.79 (0.62-1)	0.95 (0.61-1.48)	1.11 (0.81-1.52)	1.17 (0.76-1.8)	2.47 (2-3.05)	0.89 (0.58-1.37)	1.98 (1.64-2.39)
	2012-2017	0.53 (0.42-0.66)	0.58 (0.33-1.01)	0.98 (0.73-1.3)	1.82 (1.13-2.94)	1.88 (1.32-2.68)	0.97 (0.51-1.87)	1.62 (1.19-2.21)
HCV genotype		P < 0.001	P < 0.001	P < 0.001	P = 0.0035	P < 0.001	P = 0.02	P < 0.001
	1	1.0 (ref.)	1.0 (ref.)	1.0 (ref.)	1.0 (ref.)	1.0 (ref.)	1.0 (ref.)	1.0 (ref.)
	2	1.08 (0.84-1.4)	3.78 (2.53-5.63)	0.5 (0.35-0.71)	0.69 (0.47-1.02)	1.07 (0.82-1.39)	0.74 (0.5-1.08)	0.92 (0.74-1.14)
	3	1.44 (1.22-1.69)	2.6 (2.02-3.35)	1.57 (1.27-1.94)	1.42 (1.11-1.83)	1.38 (1.2-1.59)	0.94 (0.74-1.18)	1.25 (1.11-1.41)
	4	0.67 (0.54-0.84)	1.45 (0.93-2.25)	1 (0.73-1.38)	0.85 (0.57-1.27)	1.27 (1.03-1.56)	0.75 (0.52-1.07)	1.1 (0.92-1.32)
HIV status		P < 0.001	P = 0.43	P = 0.49	P = 0.21	P < 0.001	P < 0.001	P = 0.6
	Negative	1.0 (ref.)	1.0 (ref.)	1.0 (ref.)	1.0 (ref.)	1.0 (ref.)	1.0 (ref.)	1.0 (ref.)
	Positive	0.33 (0.25-0.44)	0.7 (0.39-1.26)	1.26 (0.84-1.89)	1.24 (0.8-1.91)	0.57 (0.42-0.79)	1.91 (1.39-2.62)	0.94 (0.76-1.17)
Chronic HBV infection		P = 0.72	P = 0.85	P = 0.092	P = 0.55	P = 0.016	P = 0.37	P = 0.17
	Negative	1.0 (ref.)	1.0 (ref.)	1.0 (ref.)	1.0 (ref.)	1.0 (ref.)	1.0 (ref.)	1.0 (ref.)
	Positive	0.99 (0.57-1.73)	0.93 (0.39-2.23)	1.71 (0.92-3.17)	1.26 (0.59-2.69)	0.52 (0.26-1.05)	1.44 (0.78-2.67)	0.82 (0.52-1.29)
Enrolment centre		P < 0.001	P < 0.001	P < 0.001	P = 0.2	P < 0.001	P = 0.03	P < 0.001
	Basel	1.0 (ref.)	1.0 (ref.)	1.0 (ref.)	1.0 (ref.)	1.0 (ref.)	1.0 (ref.)	1.0 (ref.)
	Bern	0.14 (0.1-0.2)	0.34 (0.22-0.52)	0.78 (0.54-1.12)	1.12 (0.67-1.87)	1.49 (1.12-1.99)	1.38 (0.91-2.09)	1.48 (1.17-1.88)
	Geneva	0.33 (0.22-0.51)	0.55 (0.34-0.91)	0.62 (0.39-1)	1.55 (0.88-2.74)	1.09 (0.76-1.58)	1.23 (0.75-2.04)	1.16 (0.86-1.56)
	Lausanne	0.25 (0.16-0.38)	0.44 (0.25-0.79)	1.13 (0.71-1.79)	1.21 (0.64-2.27)	0.92 (0.61-1.39)	0.71 (0.4-1.27)	0.86 (0.61-1.2)
	Lugano	0.33 (0.22-0.49)	0.6 (0.35-1.04)	0.56 (0.36-0.87)	1.19 (0.68-2.11)	1.16 (0.79-1.68)	1.14 (0.66-1.99)	1.11 (0.82-1.52)
	Neuch?tel	0.3 (0.2-0.45)	0.95 (0.56-1.61)	0.43 (0.27-0.7)	0.83 (0.44-1.55)	1.53 (1.11-2.12)	1.27 (0.75-2.15)	1.45 (1.1-1.9)
	St-Gall	0.37 (0.26-0.53)	1.13 (0.71-1.82)	0.44 (0.3-0.66)	1.44 (0.88-2.34)	1.61 (1.21-2.14)	1.42 (0.92-2.19)	1.53 (1.21-1.95)
	Z?rich	0.43 (0.31-0.62)	0.62 (0.42-0.91)	0.76 (0.53-1.08)	1.01 (0.62-1.66)	1.67 (1.28-2.18)	1.26 (0.84-1.89)	1.55 (1.24-1.93)
Cirrhotic at enrolment		P < 0.001	P = 0.021	X	X	P < 0.001	P < 0.001	P < 0.001
	No	1.0 (ref.)	1.0 (ref.)	X	X	1.0 (ref.)	1.0 (ref.)	1.0 (ref.)
	Yes	2.97 (2.37-3.72)	0.66 (0.5-0.87)	X	X	0.7 (0.55-0.88)	4.59 (3.7-5.69)	1.64 (1.42-1.89)
Treatment		X	X	X	P = 0.32	P < 0.001	P < 0.001	P < 0.001
	No treatment	X	X	X	1.0 (ref.)	1.0 (ref.)	1.0 (ref.)	1.0 (ref.)
	Treated with DAA	X	X	X	0.95 (0.65-1.39)	0.07 (0.04-0.14)	0.16 (0.09-0.29)	0.11 (0.07-0.16)
	Treated without DAA	X	X	X	0.9 (0.71-1.14)	1.1 (0.96-1.25)	0.74 (0.6-0.91)	0.98 (0.88-1.1)
Ever received DAA		X	P < 0.001	X	X	X	X	X
	No	X	1.0 (ref.)	X	X	X	X	X
	Yes	X	34.13 (20.84-55.9)	X	X	X	X	X

OR= odds ratio, with 95% confidence interval; HR= hazard ratio with 95% confidence interval.
ATS= antiviral treatment status; SVR= sustained virologic response; CAE= cirrhosis at enrolment; IC= incident cirrhosis during follow-up; LTFU= loss to follow-up.
DAA= direct-acting antivirals.

For the comparison of Swiss-born and foreign-born persons (unadjusted models)

		ATS	SVR	CAE	IC	LTFU	mortality	attrition
Swiss−/Foreign−born		P < 0.001	P = 0.15	P < 0.001	P = 0.4	P = 0.011	P = 0.022	P = 0.37
	Swiss−born	1.0 (ref.)	1.0 (ref.)	1.0 (ref.)	1.0 (ref.)	1.0 (ref.)	1.0 (ref.)	1.0 (ref.)
	Foreign−born	1.23 (1.09−1.39)	1.14 (0.95−1.37)	1.3 (1.12−1.51)	1.09 (0.89−1.35)	1.16 (1.04−1.31)	0.8 (0.65−0.97)	1.05 (0.95−1.16)

OR= odds ratio, with 95% confidence interval; HR= hazard ratio with 95% confidence interval.
ATS= antiviral treatment status; SVR= sustained virologic response; CAE= cirrhosis at enrolment; IC= incident cirrhosis during follow−up; LTFU= loss to follow−up.
DAA= direct−acting antivirals.

For the comparison of persons by geographic origin (unadjusted models)

		ATS	SVR	CAE	IC	LTFU	mortality	attrition
Geographic origin		P < 0.001	P = 0.16	P < 0.001	P = 0.35	P < 0.001	P = 0.0062	P = 0.57
	Switzerland	1.0 (ref.)	1.0 (ref.)	1.0 (ref.)	1.0 (ref.)	1.0 (ref.)	1.0 (ref.)	1.0 (ref.)
	Germany	1.28 (0.88−1.87)	1.27 (0.69−2.36)	1.38 (0.88−2.18)	0.64 (0.26−1.55)	1.47 (1.06−2.04)	0.52 (0.23−1.16)	1.17 (0.86−1.58)
	Italy	1.66 (1.36−2.02)	0.94 (0.72−1.23)	2.1 (1.7−2.59)	1.45 (1.08−1.97)	0.82 (0.67−1.02)	1.09 (0.83−1.43)	0.91 (0.77−1.08)
	Portugal	0.77 (0.53−1.1)	0.77 (0.42−1.38)	0.97 (0.57−1.64)	1.16 (0.6−2.26)	1.23 (0.85−1.78)	0.65 (0.31−1.38)	1.05 (0.75−1.46)
	Eastern Europe	0.98 (0.68−1.4)	1.59 (0.87−2.9)	0.78 (0.46−1.33)	0.6 (0.25−1.46)	1.59 (1.15−2.2)	0.37 (0.14−1)	1.21 (0.89−1.65)
	Southern Europe	1.2 (0.9−1.62)	1.55 (0.96−2.49)	1.07 (0.72−1.57)	1.03 (0.61−1.74)	1.35 (1.04−1.76)	0.88 (0.55−1.41)	1.2 (0.95−1.51)
	Western Europe	1.02 (0.77−1.35)	1.19 (0.76−1.86)	0.88 (0.59−1.3)	0.82 (0.48−1.41)	1.18 (0.91−1.54)	0.84 (0.53−1.31)	1.06 (0.84−1.33)
	Asia and Oceania	1.31 (0.93−1.84)	1.9 (1.07−3.36)	1.14 (0.74−1.76)	1.27 (0.71−2.27)	1.48 (1.1−1.98)	0.27 (0.1−0.73)	1.1 (0.83−1.45)
	Africa	1.05 (0.76−1.45)	1.01 (0.61−1.66)	0.94 (0.6−1.48)	1.03 (0.58−1.83)	1.04 (0.74−1.45)	0.8 (0.46−1.4)	0.97 (0.73−1.28)
	America	1.16 (0.77−1.75)	1.05 (0.56−1.98)	0.83 (0.46−1.49)	1.01 (0.47−2.13)	1.43 (1−2.05)	0.59 (0.26−1.33)	1.17 (0.84−1.62)

OR= odds ratio, with 95% confidence interval; HR= hazard ratio with 95% confidence interval.
ATS= antiviral treatment status; SVR= sustained virologic response; CAE= cirrhosis at enrolment; IC= incident cirrhosis during follow−up; LTFU= loss to follow−up.
DAA= direct−acting antivirals.
