# Supplementary material for: Impact of geographic origin on access to therapy and therapy outcomes in the Swiss Hepatitis C Cohort Study
Source: PLoS One. 2019 Jun 24;14(6):e0218706. doi: 10.1371/journal.pone.0218706 (PMC6590815; doi:10.1371/journal.pone.0218706)
Supplement: S2 Table — (PDF) [file pone.0218706.s002.pdf]

S2 Table
Characteristics of Swiss-/foreign-born persons LTFU compared to persons not LTFU

		Not LTFU	LTFU Swiss	LTFU foreign	Chi-square test p-value (LTFU Swiss versus not LTFU)	Chi-square test p-value (LTFU foreign versus not LTFU)
N		3499	801	433		
Gender	Female	1299 (37.1)	300 (37.5)	166 (38.3)	P = 0.89	P = 0.66
	Male	2200 (62.9)	501 (62.5)	267 (61.7)		
Age (y)	18-40	1164 (33.3)	432 (53.9)	202 (46.7)	P < 0.001	P < 0.001
	41-60	1866 (53.3)	312 (39)	192 (44.3)		
	≥ 61	465 (13.3)	54 (6.7)	39 (9)		
	Unknown	4 (0.1)	3 (0.4)	0 (0)		
Education	Low	715 (20.4)	185 (23.1)	119 (27.5)	P < 0.001	P = 0.0061
	Middle	2093 (59.8)	500 (62.4)	228 (52.7)		
	High	638 (18.2)	100 (12.5)	80 (18.5)		
	Unknown	53 (1.5)	16 (2)	6 (1.4)		
Employment	Unempl.	301 (8.6)	126 (15.7)	66 (15.2)	P < 0.001	P < 0.001
	Working	2125 (60.7)	452 (56.4)	277 (64)		
	Inval.	1060 (30.3)	219 (27.3)	85 (19.6)		
	Unknown	13 (0.4)	4 (0.5)	5 (1.2)		
(History of) injection drug use	Not user	1612 (46.1)	204 (25.5)	236 (54.5)	P < 0.001	P < 0.001
	Former	488 (13.9)	76 (9.5)	35 (8.1)		
	Current	1364 (39)	514 (64.2)	160 (37)		
	Unknown	35 (1)	7 (0.9)	2 (0.5)		
Alcohol consumption	Light	1846 (52.8)	428 (53.4)	257 (59.4)	P = 0.17	P = 0.0096
	Moderate	609 (17.4)	113 (14.1)	66 (15.2)		
	Excessive	792 (22.6)	200 (25)	94 (21.7)		
	Former	244 (7)	57 (7.1)	14 (3.2)		
	Unknown	8 (0.2)	3 (0.4)	2 (0.5)		
Time from diagnosis to enrolment (y)	0-2	1256 (35.9)	360 (44.9)	222 (51.3)	P < 0.001	P < 0.001
	2-6	848 (24.2)	196 (24.5)	94 (21.7)		
	6-10	639 (18.3)	129 (16.1)	65 (15)		
	10+	673 (19.2)	95 (11.9)	42 (9.7)		
	Unknown	83 (2.4)	21 (2.6)	10 (2.3)		
HCV genotype	1	1741 (49.8)	320 (40)	187 (43.2)	P < 0.001	P = 0.082
	2	280 (8)	38 (4.7)	41 (9.5)		
	3	814 (23.3)	283 (35.3)	119 (27.5)		
	4	306 (8.7)	82 (10.2)	44 (10.2)		
	Unknown	358 (10.2)	78 (9.7)	42 (9.7)		
HIV status	Negative	2452 (70.1)	622 (77.7)	318 (73.4)	P < 0.001	P = 0.12
	Positive	249 (7.1)	36 (4.5)	20 (4.6)		
	Unknown	798 (22.8)	143 (17.9)	95 (21.9)		
Chronic HBV infection	Negative	2780 (79.5)	639 (79.8)	348 (80.4)	P = 0.16	P = 0.39
	Positive	75 (2.1)	9 (1.1)	5 (1.2)		
	Unknown	644 (18.4)	153 (19.1)	80 (18.5)		
Enrolment center	Basel	264 (7.5)	52 (6.5)	23 (5.3)	P < 0.001	P = 0.0012
	Bern	694 (19.8)	179 (22.3)	66 (15.2)		
	Geneva	221 (6.3)	24 (3)	36 (8.3)		
	Lausanne	186 (5.3)	22 (2.7)	21 (4.8)		
	Lugano	348 (9.9)	43 (5.4)	39 (9)		
	Neuchâtel	241 (6.9)	68 (8.5)	42 (9.7)		
	St-Gall	635 (18.1)	181 (22.6)	63 (14.5)		
	Zürich	910 (26)	232 (29)	143 (33)		
Cirrhotic at enrolment	No	2826 (80.8)	737 (92)	390 (90.1)	P < 0.001	P < 0.001
	Yes	644 (18.4)	61 (7.6)	42 (9.7)		
	Unknown	29 (0.8)	3 (0.4)	1 (0.2)		

Characteristics of persons LTFU compared to persons not LTFU

		All	LTFU	Not LTFU	Chi-square test p-value (LTFU versus not LTFU)
N		4733 (100)	1234 (26.1)	3499 (73.9)	
Gender	Female	1765 (37.3)	466 (37.8)	1299 (37.1)	P = 0.72
	Male	2968 (62.7)	768 (62.2)	2200 (62.9)	
Age (y)	18-40	1798 (38)	634 (51.4)	1164 (33.3)	P < 0.001
	41-60	2370 (50.1)	504 (40.8)	1866 (53.3)	
	≥ 61	558 (11.8)	93 (7.5)	465 (13.3)	
	Unknown	7 (0.1)	3 (0.2)	4 (0.1)	
Education	Low	1019 (21.5)	304 (24.6)	715 (20.4)	P < 0.001
	Middle	2821 (59.6)	728 (59)	2093 (59.8)	
	High	818 (17.3)	180 (14.6)	638 (18.2)	
	Unknown	75 (1.6)	22 (1.8)	53 (1.5)	
Employment	Unempl.	493 (10.4)	192 (15.6)	301 (8.6)	P < 0.001
	Working	2854 (60.3)	729 (59.1)	2125 (60.7)	
	Inval.	1364 (28.8)	304 (24.6)	1060 (30.3)	
	Unknown	22 (0.5)	9 (0.7)	13 (0.4)	
(History of) injection drug use	Not user	2052 (43.4)	440 (35.7)	1612 (46.1)	P < 0.001
	Former	599 (12.7)	111 (9)	488 (13.9)	
	Current	2038 (43.1)	674 (54.6)	1364 (39)	
	Unknown	44 (0.9)	9 (0.7)	35 (1)	
Alcohol consumption	Light	2531 (53.5)	685 (55.5)	1846 (52.8)	P = 0.037
	Moderate	788 (16.6)	179 (14.5)	609 (17.4)	
	Excessive	1086 (22.9)	294 (23.8)	792 (22.6)	
	Former	315 (6.7)	71 (5.8)	244 (7)	
	Unknown	13 (0.3)	5 (0.4)	8 (0.2)	
Time from HCV infection to enrolment (y)	0-2	1838 (38.8)	582 (47.2)	1256 (35.9)	P < 0.001
	2-6	1138 (24)	290 (23.5)	848 (24.2)	
	6-10	833 (17.6)	194 (15.7)	639 (18.3)	
	10+	810 (17.1)	137 (11.1)	673 (19.2)	
	Unknown	114 (2.4)	31 (2.5)	83 (2.4)	
HCV genotype	1	2248 (47.5)	507 (41.1)	1741 (49.8)	P < 0.001
	2	359 (7.6)	79 (6.4)	280 (8)	
	3	1216 (25.7)	402 (32.6)	814 (23.3)	
	4	432 (9.1)	126 (10.2)	306 (8.7)	
	Unknown	478 (10.1)	120 (9.7)	358 (10.2)	
HIV status	Negative	3392 (71.7)	940 (76.2)	2452 (70.1)	P < 0.001
	Positive	305 (6.4)	56 (4.5)	249 (7.1)	
	Unknown	1036 (21.9)	238 (19.3)	798 (22.8)	
Chronic HBV infection	Negative	3767 (79.6)	987 (80)	2780 (79.5)	P = 0.035
	Positive	89 (1.9)	14 (1.1)	75 (2.1)	
	Unknown	877 (18.5)	233 (18.9)	644 (18.4)	
Test center	Basel	339 (7.2)	75 (6.1)	264 (7.5)	P < 0.001
	Bern	939 (19.8)	245 (19.9)	694 (19.8)	
	Geneva	281 (5.9)	60 (4.9)	221 (6.3)	
	Lausanne	229 (4.8)	43 (3.5)	186 (5.3)	
	Lugano	430 (9.1)	82 (6.6)	348 (9.9)	
	Neuchâtel	351 (7.4)	110 (8.9)	241 (6.9)	
	St-Gall	879 (18.6)	244 (19.8)	635 (18.1)	
	Zürich	1285 (27.1)	375 (30.4)	910 (26)	
Cirrhotic at enrolment	No	3953 (83.5)	1127 (91.3)	2826 (80.8)	P < 0.001
	Yes	747 (15.8)	103 (8.3)	644 (18.4)	
	Unknown	33 (0.7)	4 (0.3)	29 (0.8)	
Swiss-born	Yes	3196 (67.5)	801 (64.9)	2395 (68.4)	P = 0.025
	No	1537 (32.5)	433 (35.1)	1104 (31.6)	
Geographic origin	Switzerland	3196 (67.5)	801 (64.9)	2395 (68.4)	P < 0.001
	Germany	112 (2.4)	37 (3)	75 (2.1)	
	Italy	463 (9.8)	96 (7.8)	367 (10.5)	
	Portugal	105 (2.2)	29 (2.4)	76 (2.2)	
	Eastern Europe	111 (2.3)	38 (3.1)	73 (2.1)	
	Southern Europe	179 (3.8)	59 (4.8)	120 (3.4)	
	Western Europe	199 (4.2)	60 (4.9)	139 (4)	
	Asia and Oceania	137 (2.9)	47 (3.8)	90 (2.6)	
	Africa	141 (3)	36 (2.9)	105 (3)	
	America	90 (1.9)	31 (2.5)	59 (1.7)	
