# Supplementary material for: Impact of geographic origin on access to therapy and therapy outcomes in the Swiss Hepatitis C Cohort Study
Source: PLoS One. 2019 Jun 24;14(6):e0218706. doi: 10.1371/journal.pone.0218706 (PMC6590815; doi:10.1371/journal.pone.0218706)
Supplement: S3 Table — (PDF) [file pone.0218706.s003.pdf]

S3 Table

Sensitivity analysis: comparison of Swiss-born and foreign-born persons, with calculation of missing values by multiple imputation

		ATS	SVR	CAE	IC	LTFU	mortality	attrition
Swiss-/Foreign-born origin		P = 0.43	P = 0.18	P = 0.02	NA	NA	NA	NA
	Swiss-born	1.0 (ref.)	1.0 (ref.)	1.0 (ref.)	1.0 (ref.)	1.0 (ref.)	1.0 (ref.)	1.0 (ref.)
	Foreign-born	1.06 (0.92-1.21)	1.18 (0.94-1.48)	1.22 (1.02-1.45)	0.99 (0.79-1.25)	1.34 (1.18-1.52)	0.76 (0.61-0.94)	1.14 (1.02-1.27)
Gender		P < 0.001	P = 0.11	P < 0.001	NA	NA	NA	NA
	Female	1.0 (ref.)	1.0 (ref.)	1.0 (ref.)	1.0 (ref.)	1.0 (ref.)	1.0 (ref.)	1.0 (ref.)
	Male	1.47 (1.29-1.67)	0.88 (0.71-1.1)	1.62 (1.36-1.93)	1.41 (1.13-1.77)	1.01 (0.9-1.14)	1.3 (1.06-1.59)	1.08 (0.98-1.2)
Age (y)		P < 0.001	P = 0.2	P < 0.001	NA	NA	NA	NA
	18-40	1.0 (ref.)	1.0 (ref.)	1.0 (ref.)	1.0 (ref.)	1.0 (ref.)	1.0 (ref.)	1.0 (ref.)
	41-60	1.7 (1.48-1.95)	0.66 (0.52-0.84)	4.8 (3.77-6.12)	2.71 (2.08-3.52)	0.73 (0.65-0.83)	1.72 (1.36-2.18)	0.86 (0.77-0.96)
	≥ 61	1.08 (0.85-1.38)	0.61 (0.41-0.93)	8.97 (6.47-12.43)	5.1 (3.42-7.61)	0.76 (0.58-1)	3.24 (2.26-4.66)	1.16 (0.95-1.42)
Education		P = 0.088	P = 0.017	P = 0.014	NA	NA	NA	NA
	Low	0.91 (0.78-1.07)	1.03 (0.79-1.35)	1.13 (0.92-1.38)	1.34 (1.05-1.73)	1.14 (1-1.32)	1.09 (0.87-1.36)	1.11 (0.98-1.24)
	Middle	1.0 (ref.)	1.0 (ref.)	1.0 (ref.)	1.0 (ref.)	1.0 (ref.)	1.0 (ref.)	1.0 (ref.)
	High	0.92 (0.78-1.09)	1.44 (1.08-1.91)	0.91 (0.73-1.12)	0.98 (0.74-1.29)	1 (0.84-1.18)	0.96 (0.74-1.25)	0.99 (0.86-1.14)
Employment		P < 0.001	P = 0.16	P < 0.001	NA	NA	NA	NA
	Unempl.	1.0 (ref.)	1.0 (ref.)	1.0 (ref.)	1.0 (ref.)	1.0 (ref.)	1.0 (ref.)	1.0 (ref.)
	Working	1.34 (1.09-1.65)	0.79 (0.54-1.16)	0.97 (0.71-1.33)	0.91 (0.62-1.33)	0.7 (0.59-0.83)	0.63 (0.46-0.86)	0.68 (0.59-0.79)
	Inval.	1.07 (0.85-1.34)	0.53 (0.35-0.81)	1.44 (1.03-2)	1.03 (0.69-1.56)	0.74 (0.61-0.89)	1.13 (0.83-1.55)	0.84 (0.72-0.99)
(History of) injection drug use		P < 0.001	P < 0.001	P = 0.081	NA	NA	NA	NA
	Not user	1.0 (ref.)	1.0 (ref.)	1.0 (ref.)	1.0 (ref.)	1.0 (ref.)	1.0 (ref.)	1.0 (ref.)
	Former	0.62 (0.5-0.76)	0.86 (0.56-1.3)	0.58 (0.44-0.76)	1.03 (0.71-1.5)	1.18 (0.93-1.5)	1.36 (0.93-1.99)	1.2 (0.99-1.47)
	Current	0.61 (0.52-0.72)	1.03 (0.8-1.34)	0.57 (0.46-0.72)	0.76 (0.58-1.01)	1.35 (1.16-1.57)	0.92 (0.71-1.18)	1.19 (1.05-1.36)
Alcohol consumption		P < 0.001	P = 0.018	P < 0.001	NA	NA	NA	NA
	Light	1.0 (ref.)	1.0 (ref.)	1.0 (ref.)	1.0 (ref.)	1.0 (ref.)	1.0 (ref.)	1.0 (ref.)
	Moderate	1.14 (0.96-1.36)	1.17 (0.87-1.56)	1.23 (0.98-1.54)	1.13 (0.84-1.51)	0.89 (0.75-1.05)	1.18 (0.89-1.56)	0.95 (0.82-1.09)
	Excessive	0.81 (0.69-0.95)	0.78 (0.59-1.03)	2.83 (2.3-3.48)	1.69 (1.29-2.22)	0.95 (0.82-1.11)	2.01 (1.6-2.54)	1.15 (1.02-1.3)
	Former	1.09 (0.85-1.41)	1.07 (0.68-1.69)	2.29 (1.67-3.13)	1.48 (0.98-2.23)	0.78 (0.6-1.01)	1.5 (1.07-2.13)	0.93 (0.76-1.14)
Time from diagnosis to enrolment (y)		P < 0.001	P < 0.001	P = 0.012	NA	NA	NA	NA
	0-2	1.0 (ref.)	1.0 (ref.)	1.0 (ref.)	1.0 (ref.)	1.0 (ref.)	1.0 (ref.)	1.0 (ref.)
	2-6	1.39 (1.19-1.63)	0.63 (0.48-0.83)	1.06 (0.85-1.33)	1.15 (0.88-1.5)	0.82 (0.71-0.95)	0.81 (0.64-1.03)	0.81 (0.72-0.92)
	6-10	1.5 (1.25-1.79)	0.68 (0.51-0.91)	1.18 (0.93-1.49)	1.26 (0.94-1.68)	0.78 (0.66-0.92)	1 (0.78-1.28)	0.84 (0.73-0.96)
	10+	1.47 (1.23-1.75)	0.55 (0.41-0.74)	1.31 (1.06-1.62)	1.27 (0.95-1.7)	0.69 (0.57-0.83)	0.94 (0.73-1.23)	0.75 (0.64-0.87)
Calendar year of enrolment		P < 0.001	P = 0.65	P = 0.97	NA	NA	NA	NA
	2000-2003	1.0 (ref.)	1.0 (ref.)	1.0 (ref.)	1.0 (ref.)	1.0 (ref.)	1.0 (ref.)	1.0 (ref.)
	2004-2007	0.79 (0.68-0.92)	0.97 (0.77-1.24)	1.03 (0.83-1.27)	1.3 (1.03-1.65)	1.58 (1.39-1.8)	1.12 (0.91-1.37)	1.45 (1.3-1.61)
	2008-2011	0.8 (0.64-0.99)	0.97 (0.64-1.45)	1.05 (0.78-1.41)	1.29 (0.87-1.91)	2.35 (1.93-2.87)	1 (0.7-1.44)	1.94 (1.64-2.31)
	2012-2017	0.58 (0.48-0.7)	0.73 (0.46-1.17)	1.06 (0.83-1.35)	1.84 (1.2-2.81)	1.94 (1.46-2.6)	1.25 (0.79-2)	1.78 (1.39-2.27)
HCV genotype		P < 0.001	P < 0.001	P < 0.001	NA	NA	NA	NA
	1	1.0 (ref.)	1.0 (ref.)	1.0 (ref.)	1.0 (ref.)	1.0 (ref.)	1.0 (ref.)	1.0 (ref.)
	2	1.11 (0.87-1.41)	3.43 (2.33-5.03)	0.51 (0.37-0.72)	0.7 (0.47-1.03)	1.05 (0.82-1.35)	0.73 (0.51-1.05)	0.92 (0.75-1.13)
	3	1.48 (1.26-1.73)	2.5 (1.96-3.2)	1.58 (1.29-1.93)	1.42 (1.1-1.82)	1.39 (1.21-1.59)	0.93 (0.74-1.16)	1.25 (1.11-1.41)
	4	0.7 (0.56-0.87)	1.31 (0.86-1.98)	0.99 (0.73-1.33)	0.86 (0.58-1.28)	1.26 (1.04-1.54)	0.76 (0.54-1.09)	1.11 (0.94-1.31)
HIV status		P < 0.001	P = 0.66	P = 0.41	NA	NA	NA	NA
	Negative	1.0 (ref.)	1.0 (ref.)	1.0 (ref.)	1.0 (ref.)	1.0 (ref.)	1.0 (ref.)	1.0 (ref.)
	Positive	0.34 (0.26-0.45)	0.8 (0.45-1.41)	1.24 (0.85-1.8)	1.09 (0.72-1.65)	0.56 (0.42-0.75)	1.65 (1.22-2.23)	0.86 (0.7-1.06)
Chronic HBV infection		P = 0.89	P = 0.27	P = 0.13	NA	NA	NA	NA
	Negative	1.0 (ref.)	1.0 (ref.)	1.0 (ref.)	1.0 (ref.)	1.0 (ref.)	1.0 (ref.)	1.0 (ref.)
	Positive	0.84 (0.53-1.32)	0.95 (0.43-2.13)	1.73 (1.01-2.96)	1.31 (0.62-2.78)	0.65 (0.38-1.11)	1.47 (0.87-2.46)	0.85 (0.58-1.25)
Enrolment centre		P < 0.001	P < 0.001	P < 0.001	NA	NA	NA	NA
	Basel	1.0 (ref.)	1.0 (ref.)	1.0 (ref.)	1.0 (ref.)	1.0 (ref.)	1.0 (ref.)	1.0 (ref.)
	Bern	0.15 (0.11-0.21)	0.34 (0.23-0.51)	0.82 (0.58-1.15)	1.28 (0.78-2.07)	1.51 (1.15-1.97)	1.27 (0.86-1.86)	1.42 (1.14-1.76)
	Geneva	0.36 (0.24-0.52)	0.59 (0.37-0.94)	0.69 (0.45-1.06)	1.6 (0.94-2.74)	1.07 (0.76-1.52)	1.15 (0.73-1.83)	1.09 (0.83-1.44)
	Lausanne	0.34 (0.23-0.51)	0.46 (0.27-0.77)	1.17 (0.77-1.77)	1.35 (0.74-2.46)	0.96 (0.66-1.4)	0.72 (0.42-1.21)	0.88 (0.65-1.2)
	Lugano	0.38 (0.27-0.53)	0.64 (0.39-1.03)	0.7 (0.48-1.02)	1.21 (0.71-2.05)	1.29 (0.93-1.78)	1.2 (0.74-1.94)	1.22 (0.93-1.59)
	Neuchâtel	0.3 (0.21-0.44)	0.96 (0.58-1.58)	0.41 (0.26-0.64)	0.86 (0.47-1.57)	1.47 (1.08-1.99)	1.19 (0.74-1.93)	1.35 (1.05-1.74)
	St-Gall	0.37 (0.27-0.51)	1.22 (0.78-1.91)	0.45 (0.31-0.64)	1.46 (0.91-2.33)	1.59 (1.22-2.08)	1.37 (0.92-2.02)	1.47 (1.18-1.83)
	Zürich	0.43 (0.31-0.58)	0.68 (0.47-0.98)	0.73 (0.53-1.01)	0.99 (0.62-1.59)	1.69 (1.32-2.18)	1.2 (0.83-1.74)	1.51 (1.23-1.85)
Cirrhotic at enrolment		P < 0.001	P = 0.036	X	X	NA	NA	NA
	No	1.0 (ref.)	1.0 (ref.)	X	X	1.0 (ref.)	1.0 (ref.)	1.0 (ref.)
	Yes	2.95 (2.42-3.59)	0.7 (0.54-0.9)	X	X	0.71 (0.57-0.88)	4.54 (3.73-5.53)	1.65 (1.45-1.88)
Treatment		X	X	X	NA	NA	NA	NA
	No treatment	X	X	X	1.0 (ref.)	1.0 (ref.)	1.0 (ref.)	1.0 (ref.)
	Treated with DAA	X	X	X	1.08 (0.76-1.52)	0.08 (0.04-0.14)	0.17 (0.1-0.27)	0.12 (0.08-0.17)
	Treated without DAA	X	X	X	0.95 (0.75-1.19)	1.02 (0.91-1.16)	0.7 (0.58-0.85)	0.92 (0.83-1.02)
Ever received DAA		X	P < 0.001	X	X	X	X	X
	No	X	1.0 (ref.)	X	X	X	X	X
	Yes	X	26.35 (17.23-40.29)	X	X	X	X	X

ATS= antiviral treatment status; SVR= sustained virologic response; CAE= cirrhosis at enrolment; IC= incident cirrhosis during follow-up; LTFU= loss to follow-up.
DAA= direct-acting antivirals.

Sensitivity analysis: comparison of Swiss-born and foreign-born persons, with removal of persons with missing values

		ATS	SVR	CAE	IC	LTFU	mortality	attrition
Swiss-/Foreign-born		P = 0.78	P = 0.091	P = 0.022	P = 0.69	P < 0.001	P = 0.023	P = 0.072
	Swiss-Born	1.0 (ref.)	1.0 (ref.)	1.0 (ref.)	1.0 (ref.)	1.0 (ref.)	1.0 (ref.)	1.0 (ref.)
	Foreign-born	0.99 (0.82-1.2)	1.29 (0.97-1.72)	1.32 (1.04-1.67)	1.06 (0.79-1.41)	1.35 (1.14-1.61)	0.73 (0.55-0.96)	1.12 (0.97-1.3)
Gender		P < 0.001	P = 0.094	P < 0.001	P = 0.071	P = 0.75	P < 0.001	P = 0.12
	Female	1.0 (ref.)	1.0 (ref.)	1.0 (ref.)	1.0 (ref.)	1.0 (ref.)	1.0 (ref.)	1.0 (ref.)
	Male	1.4 (1.17-1.67)	0.87 (0.66-1.15)	1.61 (1.26-2.05)	1.19 (0.9-1.58)	0.97 (0.83-1.14)	1.2 (0.92-1.56)	1.02 (0.89-1.17)
Age (y)		P < 0.001	P = 0.04	P < 0.001	P < 0.001	P < 0.001	P < 0.001	P = 0.0018
	18-40	1.0 (ref.)	1.0 (ref.)	1.0 (ref.)	1.0 (ref.)	1.0 (ref.)	1.0 (ref.)	1.0 (ref.)
	41-60	1.65 (1.37-1.99)	0.75 (0.56-1)	5.36 (3.98-7.24)	2.69 (1.97-3.66)	0.72 (0.61-0.85)	1.64 (1.23-2.2)	0.85 (0.74-0.98)
	≥ 61	1.08 (0.75-1.55)	0.56 (0.33-0.95)	11.17 (7.19-17.34)	4.11 (2.41-7.02)	0.82 (0.56-1.21)	3.44 (2.18-5.45)	1.32 (1.01-1.74)
Education		P = 0.074	P = 0.06	P = 0.27	P = 0.44	P = 0.13	P = 0.11	P = 0.0061
	Low	0.93 (0.76-1.13)	0.81 (0.58-1.11)	1.1 (0.84-1.44)	1.19 (0.86-1.64)	1.15 (0.96-1.38)	1.17 (0.88-1.54)	1.14 (0.98-1.33)
	Middle	1.0 (ref.)	1.0 (ref.)	1.0 (ref.)	1.0 (ref.)	1.0 (ref.)	1.0 (ref.)	1.0 (ref.)
	High	0.92 (0.72-1.18)	1.36 (0.94-1.97)	0.97 (0.72-1.3)	1.15 (0.81-1.64)	1.06 (0.84-1.34)	1.03 (0.72-1.46)	1.06 (0.87-1.29)
Employment		P < 0.001	P = 0.092	P = 0.099	P = 0.28	P < 0.001	P < 0.001	P < 0.001
	Unempl.	1.0 (ref.)	1.0 (ref.)	1.0 (ref.)	1.0 (ref.)	1.0 (ref.)	1.0 (ref.)	1.0 (ref.)
	Working	1.34 (1.01-1.77)	0.7 (0.44-1.11)	1.09 (0.72-1.65)	0.83 (0.52-1.32)	0.61 (0.5-0.76)	0.69 (0.46-1.04)	0.63 (0.52-0.76)
	Inval.	1.08 (0.8-1.47)	0.49 (0.3-0.82)	1.32 (0.85-2.05)	0.86 (0.52-1.42)	0.61 (0.47-0.78)	1.25 (0.82-1.88)	0.77 (0.62-0.94)
(History of) injection drug use		P < 0.001	P = 0.012	P = 0.23	P = 0.17	P = 0.051	P = 0.2	P = 0.035
	Not user	1.0 (ref.)	1.0 (ref.)	1.0 (ref.)	1.0 (ref.)	1.0 (ref.)	1.0 (ref.)	1.0 (ref.)
	Former	0.6 (0.44-0.82)	1.08 (0.61-1.91)	0.63 (0.43-0.94)	0.97 (0.59-1.59)	1.26 (0.9-1.77)	1.17 (0.7-1.97)	1.16 (0.87-1.54)
	Current	0.63 (0.51-0.79)	1.2 (0.87-1.65)	0.54 (0.41-0.71)	0.71 (0.51-1)	1.34 (1.1-1.63)	0.86 (0.63-1.17)	1.15 (0.97-1.35)
Alcohol consumption		P = 0.037	P = 0.1	P < 0.001	P = 0.01	P = 0.13	P < 0.001	P = 0.0034
	Light	1.0 (ref.)	1.0 (ref.)	1.0 (ref.)	1.0 (ref.)	1.0 (ref.)	1.0 (ref.)	1.0 (ref.)
	Moderate	1.3 (1.02-1.65)	1.03 (0.72-1.48)	1.42 (1.05-1.94)	1.3 (0.9-1.87)	0.84 (0.68-1.05)	1.16 (0.81-1.65)	0.89 (0.73-1.07)
	Excessive	0.88 (0.71-1.09)	0.65 (0.46-0.91)	3.32 (2.52-4.38)	1.79 (1.26-2.52)	0.97 (0.8-1.17)	1.98 (1.47-2.66)	1.16 (0.99-1.36)
	Former	1.07 (0.76-1.51)	0.69 (0.39-1.2)	2.39 (1.56-3.67)	1.69 (1.02-2.79)	0.77 (0.55-1.07)	1.77 (1.16-2.69)	0.99 (0.77-1.28)
Time from diagnosis to enrolment (y)		P < 0.001	P < 0.001	P = 0.016	P = 0.072	P < 0.001	P = 0.3	P < 0.001
	0-2	1.0 (ref.)	1.0 (ref.)	1.0 (ref.)	1.0 (ref.)	1.0 (ref.)	1.0 (ref.)	1.0 (ref.)
	2-6	1.33 (1.08-1.64)	0.61 (0.44-0.85)	0.99 (0.74-1.33)	1.12 (0.8-1.58)	0.77 (0.64-0.93)	0.78 (0.57-1.05)	0.78 (0.66-0.91)
	6-10	1.75 (1.37-2.23)	0.69 (0.49-0.98)	1.25 (0.92-1.69)	1.41 (0.99-2.02)	0.78 (0.63-0.96)	1.04 (0.77-1.41)	0.84 (0.7-0.99)
	10+	1.6 (1.24-2.06)	0.58 (0.4-0.84)	1.49 (1.11-2.01)	1.48 (1.02-2.14)	0.59 (0.45-0.77)	0.85 (0.6-1.19)	0.66 (0.54-0.82)
Calendar year of enrolment		P < 0.001	P = 0.15	P = 0.63	P = 0.077	P < 0.001	P = 0.7	P < 0.001
	2000-2003	1.0 (ref.)	1.0 (ref.)	1.0 (ref.)	1.0 (ref.)	1.0 (ref.)	1.0 (ref.)	1.0 (ref.)
	2004-2007	0.76 (0.63-0.93)	0.77 (0.57-1.02)	1.14 (0.88-1.48)	1.27 (0.95-1.7)	1.6 (1.36-1.88)	1.13 (0.88-1.46)	1.46 (1.27-1.67)
	2008-2011	0.78 (0.57-1.06)	0.94 (0.55-1.61)	0.91 (0.61-1.35)	1.07 (0.62-1.84)	2.55 (1.95-3.33)	1.17 (0.71-1.93)	2.16 (1.71-2.73)
	2012-2017	0.45 (0.33-0.6)	0.56 (0.28-1.1)	1.02 (0.7-1.47)	2.06 (1.13-3.75)	1.65 (1.02-2.67)	0.84 (0.34-2.06)	1.42 (0.93-2.16)
HCV genotype		P < 0.001	P < 0.001	P < 0.001	P = 0.021	P < 0.001	P < 0.001	P < 0.001
	1	1.0 (ref.)	1.0 (ref.)	1.0 (ref.)	1.0 (ref.)	1.0 (ref.)	1.0 (ref.)	1.0 (ref.)
	2	0.85 (0.6-1.21)	4.06 (2.46-6.71)	0.41 (0.25-0.66)	0.67 (0.4-1.09)	0.9 (0.62-1.3)	0.48 (0.28-0.84)	0.7 (0.51-0.94)
	3	1.34 (1.1-1.63)	2.99 (2.22-4.01)	1.6 (1.25-2.05)	1.36 (1.01-1.83)	1.43 (1.22-1.69)	0.87 (0.67-1.13)	1.25 (1.09-1.44)
	4	0.67 (0.51-0.87)	1.61 (0.98-2.64)	1 (0.69-1.44)	0.77 (0.48-1.25)	1.11 (0.86-1.43)	0.71 (0.47-1.06)	0.98 (0.79-1.22)
HIV status		P < 0.001	P = 0.25	P = 0.43	P = 0.16	P = 0.0013	P < 0.001	P = 0.77
	Negative	1.0 (ref.)	1.0 (ref.)	1.0 (ref.)	1.0 (ref.)	1.0 (ref.)	1.0 (ref.)	1.0 (ref.)
	Positive	0.28 (0.2-0.38)	0.59 (0.31-1.13)	1.29 (0.83-1.99)	1.28 (0.8-2.07)	0.61 (0.43-0.86)	2.01 (1.43-2.82)	1 (0.8-1.27)
Chronic HBV infection		P = 0.9	P = 0.98	P = 0.15	P = 0.75	P = 0.052	P = 0.38	P = 0.33
	Negative	1.0 (ref.)	1.0 (ref.)	1.0 (ref.)	1.0 (ref.)	1.0 (ref.)	1.0 (ref.)	1.0 (ref.)
	Positive	0.95 (0.51-1.74)	0.97 (0.38-2.45)	1.67 (0.86-3.26)	0.81 (0.3-2.19)	0.53 (0.25-1.11)	1.41 (0.74-2.7)	0.82 (0.51-1.34)
Enrolment centre		P < 0.001	P < 0.001	P < 0.001	P = 0.41	P = 0.0013	P = 0.026	P < 0.001
	Basel	1.0 (ref.)	1.0 (ref.)	1.0 (ref.)	1.0 (ref.)	1.0 (ref.)	1.0 (ref.)	1.0 (ref.)
	Bern	0.14 (0.09-0.22)	0.34 (0.21-0.56)	0.84 (0.53-1.32)	1.06 (0.59-1.91)	1.54 (1.09-2.18)	1.11 (0.7-1.76)	1.41 (1.07-1.86)
	Geneva	0.3 (0.18-0.51)	0.8 (0.43-1.49)	0.58 (0.32-1.06)	1.53 (0.79-2.97)	1.11 (0.7-1.76)	1.14 (0.64-2.04)	1.13 (0.79-1.63)
	Lausanne	0.22 (0.13-0.38)	0.64 (0.33-1.21)	1.24 (0.72-2.14)	1.21 (0.6-2.44)	1.08 (0.68-1.71)	0.5 (0.26-0.97)	0.85 (0.58-1.25)
	Lugano	0.28 (0.17-0.46)	0.65 (0.33-1.29)	0.52 (0.29-0.95)	1.13 (0.57-2.23)	0.96 (0.59-1.55)	0.72 (0.34-1.51)	0.83 (0.56-1.25)
	Neuchâtel	0.26 (0.16-0.41)	1.04 (0.59-1.85)	0.52 (0.31-0.88)	0.75 (0.37-1.5)	1.57 (1.08-2.29)	0.88 (0.49-1.57)	1.3 (0.95-1.77)
	St-Gall	0.35 (0.23-0.54)	1.2 (0.71-2.03)	0.53 (0.33-0.84)	1.33 (0.76-2.32)	1.61 (1.14-2.26)	1.03 (0.64-1.64)	1.36 (1.03-1.8)
	Zürich	0.4 (0.26-0.61)	0.69 (0.44-1.08)	1.07 (0.7-1.62)	1.02 (0.59-1.78)	1.71 (1.24-2.36)	0.98 (0.64-1.51)	1.41 (1.09-1.83)
Cirrhotic at enrolment		P < 0.001	P = 0.015	X	X	P < 0.001	P < 0.001	P < 0.001
	No	1.0 (ref.)	1.0 (ref.)	X	X	1.0 (ref.)	1.0 (ref.)	1.0 (ref.)
	Yes	2.88 (2.18-3.81)	0.62 (0.44-0.86)	X	X	0.7 (0.53-0.91)	4.49 (3.49-5.79)	1.65 (1.39-1.95)
Treatment		X	X	X	P = 0.55	P < 0.001	P < 0.001	P < 0.001
	No treatment	X	X	X	1.0 (ref.)	1.0 (ref.)	1.0 (ref.)	1.0 (ref.)
	Treated with DAA	X	X	X	0.71 (0.42-1.19)	0.03 (0.01-0.1)	0.19 (0.1-0.36)	0.1 (0.06-0.16)
	Treated without DAA	X	X	X	0.9 (0.68-1.19)	1.02 (0.87-1.2)	0.72 (0.56-0.92)	0.93 (0.81-1.06)
Ever received DAA		X	P < 0.001	X	X	X	X	X
	No	X	1.0 (ref.)	X	X	X	X	X
	Yes	X	25.82 (14.95-44.62)	X	X	X	X	X

ATS= antiviral treatment status; SVR= sustained virologic response; CAE= cirrhosis at enrolment; IC= incident cirrhosis during follow-up; LTFU= loss to follow-up.
DAA= direct-acting antivirals.

Sensitivity analysis: comparison of persons by geographic origin, with calculation of missing values by multiple imputation

		ATS	SVR	CAE	IC	LTFU	mortality	attrition
Geographic origin		P = 0.33	P = 0.12	P = 0.021	NA	NA	NA	NA
	Switzerland	1.0 (ref.)	1.0 (ref.)	1.0 (ref.)	1.0 (ref.)	1.0 (ref.)	1.0 (ref.)	1.0 (ref.)
	Germany	0.91 (0.6-1.38)	1.15 (0.57-2.3)	1.33 (0.81-2.17)	0.6 (0.25-1.47)	1.73 (1.24-2.42)	0.46 (0.2-1.04)	1.25 (0.92-1.7)
	Italy	1.23 (0.99-1.54)	1.06 (0.75-1.49)	1.59 (1.25-2.03)	1.1 (0.79-1.54)	1.08 (0.86-1.36)	0.76 (0.56-1.03)	0.98 (0.82-1.17)
	Portugal	0.77 (0.51-1.16)	0.5 (0.24-1.05)	0.88 (0.49-1.59)	1.12 (0.56-2.22)	1.18 (0.8-1.74)	0.92 (0.43-1.98)	1.1 (0.78-1.56)
	Eastern Europe	0.9 (0.61-1.34)	1.38 (0.67-2.86)	1.01 (0.57-1.81)	0.74 (0.3-1.82)	1.9 (1.35-2.68)	0.49 (0.18-1.31)	1.52 (1.1-2.08)
	Southern Europe	1.08 (0.78-1.5)	1.73 (0.99-3.03)	1.17 (0.76-1.78)	0.88 (0.51-1.52)	1.55 (1.18-2.03)	0.98 (0.6-1.59)	1.33 (1.05-1.68)
	Western Europe	0.95 (0.7-1.29)	1.29 (0.77-2.15)	0.84 (0.54-1.28)	0.76 (0.44-1.31)	1.29 (0.98-1.68)	0.87 (0.55-1.37)	1.14 (0.9-1.43)
	Asia/Oceania	1.27 (0.86-1.85)	1.93 (1.01-3.71)	1.33 (0.82-2.15)	1.42 (0.78-2.58)	1.4 (1.03-1.9)	0.31 (0.11-0.84)	1.08 (0.81-1.44)
	Africa	1.06 (0.73-1.53)	0.91 (0.49-1.71)	0.94 (0.57-1.55)	1.13 (0.62-2.07)	1.09 (0.76-1.55)	1.1 (0.61-2)	1.07 (0.79-1.45)
	America	1.02 (0.66-1.59)	1.16 (0.56-2.39)	0.86 (0.46-1.61)	1.17 (0.54-2.51)	1.7 (1.18-2.45)	0.8 (0.35-1.82)	1.38 (0.99-1.93)
Gender		P < 0.001	P = 0.11	P < 0.001	NA	NA	NA	NA
	Female	1.0 (ref.)	1.0 (ref.)	1.0 (ref.)	1.0 (ref.)	1.0 (ref.)	1.0 (ref.)	1.0 (ref.)
	Male	1.45 (1.27-1.65)	0.9 (0.72-1.13)	1.57 (1.31-1.88)	1.37 (1.09-1.71)	1.04 (0.92-1.17)	1.29 (1.05-1.58)	1.1 (0.99-1.22)
Age (y)		P < 0.001	P = 0.2	P < 0.001	NA	NA	NA	NA
	18-40	1.0 (ref.)	1.0 (ref.)	1.0 (ref.)	1.0 (ref.)	1.0 (ref.)	1.0 (ref.)	1.0 (ref.)
	41-60	1.69 (1.47-1.95)	0.67 (0.53-0.85)	4.74 (3.72-6.05)	2.72 (2.08-3.54)	0.75 (0.66-0.85)	1.72 (1.36-2.17)	0.87 (0.78-0.97)
	≥ 61	1.06 (0.83-1.35)	0.63 (0.41-0.96)	8.48 (6.09-11.81)	5.13 (3.42-7.71)	0.81 (0.61-1.06)	3.26 (2.26-4.71)	1.2 (0.98-1.48)
Education		P = 0.088	P = 0.017	P = 0.014	NA	NA	NA	NA
	Low	0.91 (0.78-1.06)	1.06 (0.81-1.4)	1.1 (0.9-1.35)	1.3 (1-1.68)	1.16 (1.01-1.33)	1.09 (0.87-1.36)	1.11 (0.99-1.25)
	Middle	1.0 (ref.)	1.0 (ref.)	1.0 (ref.)	1.0 (ref.)	1.0 (ref.)	1.0 (ref.)	1.0 (ref.)
	High	0.93 (0.79-1.1)	1.41 (1.06-1.89)	0.94 (0.76-1.17)	1 (0.76-1.33)	0.97 (0.81-1.15)	0.98 (0.75-1.28)	0.97 (0.84-1.12)
Employment		P < 0.001	P = 0.16	P < 0.001	NA	NA	NA	NA
	Unempl.	1.0 (ref.)	1.0 (ref.)	1.0 (ref.)	1.0 (ref.)	1.0 (ref.)	1.0 (ref.)	1.0 (ref.)
	Working	1.35 (1.1-1.66)	0.81 (0.55-1.18)	0.96 (0.7-1.32)	0.91 (0.62-1.34)	0.7 (0.59-0.83)	0.63 (0.46-0.86)	0.68 (0.59-0.79)
	Inval.	1.06 (0.85-1.34)	0.53 (0.35-0.81)	1.42 (1.02-1.98)	1.04 (0.69-1.56)	0.74 (0.61-0.89)	1.14 (0.83-1.56)	0.84 (0.72-0.99)
(History of) injection drug use		P < 0.001	P < 0.001	P = 0.081	NA	NA	NA	NA
	Not user	1.0 (ref.)	1.0 (ref.)	1.0 (ref.)	1.0 (ref.)	1.0 (ref.)	1.0 (ref.)	1.0 (ref.)
	Former	0.61 (0.5-0.76)	0.86 (0.57-1.31)	0.57 (0.43-0.75)	1.04 (0.71-1.52)	1.2 (0.94-1.53)	1.38 (0.94-2.03)	1.22 (1-1.49)
	Current	0.61 (0.51-0.72)	1.04 (0.8-1.35)	0.57 (0.46-0.71)	0.78 (0.59-1.03)	1.37 (1.17-1.6)	0.92 (0.72-1.19)	1.2 (1.05-1.37)
Alcohol consumption		P < 0.001	P = 0.018	P < 0.001	NA	NA	NA	NA
	Light	1.0 (ref.)	1.0 (ref.)	1.0 (ref.)	1.0 (ref.)	1.0 (ref.)	1.0 (ref.)	1.0 (ref.)
	Moderate	1.15 (0.97-1.37)	1.19 (0.89-1.59)	1.23 (0.98-1.55)	1.14 (0.85-1.52)	0.89 (0.75-1.05)	1.18 (0.89-1.56)	0.95 (0.82-1.1)
	Excessive	0.82 (0.7-0.96)	0.8 (0.6-1.06)	2.87 (2.33-3.54)	1.74 (1.32-2.28)	0.94 (0.81-1.09)	2.01 (1.59-2.54)	1.14 (1.01-1.3)
	Former	1.1 (0.85-1.42)	1.11 (0.7-1.74)	2.31 (1.69-3.17)	1.5 (1-2.27)	0.77 (0.6-1)	1.51 (1.07-2.14)	0.93 (0.76-1.14)
Time from diagnosis to enrolment (y)		P < 0.001	P < 0.001	P = 0.012	NA	NA	NA	NA
	0-2	1.0 (ref.)	1.0 (ref.)	1.0 (ref.)	1.0 (ref.)	1.0 (ref.)	1.0 (ref.)	1.0 (ref.)
	2-6	1.4 (1.19-1.64)	0.63 (0.48-0.83)	1.07 (0.86-1.34)	1.16 (0.88-1.52)	0.82 (0.71-0.95)	0.8 (0.63-1.02)	0.81 (0.72-0.92)
	6-10	1.5 (1.26-1.79)	0.68 (0.51-0.91)	1.19 (0.94-1.51)	1.27 (0.95-1.69)	0.78 (0.66-0.93)	0.99 (0.77-1.26)	0.84 (0.73-0.96)
	10+	1.46 (1.23-1.75)	0.55 (0.41-0.74)	1.29 (1.04-1.6)	1.29 (0.96-1.73)	0.69 (0.57-0.83)	0.95 (0.73-1.23)	0.75 (0.64-0.87)
Calendar year of enrolment		P < 0.001	P = 0.66	P = 0.89	NA	NA	NA	NA
	2000-2003	1.0 (ref.)	1.0 (ref.)	1.0 (ref.)	1.0 (ref.)	1.0 (ref.)	1.0 (ref.)	1.0 (ref.)
	2004-2007	0.8 (0.68-0.93)	0.97 (0.76-1.23)	1.04 (0.84-1.29)	1.3 (1.03-1.65)	1.57 (1.38-1.79)	1.11 (0.91-1.36)	1.44 (1.3-1.61)
	2008-2011	0.81 (0.65-1.01)	0.95 (0.63-1.44)	1.07 (0.79-1.43)	1.28 (0.86-1.9)	2.32 (1.91-2.83)	1 (0.7-1.45)	1.93 (1.62-2.29)
	2012-2017	0.58 (0.48-0.71)	0.73 (0.46-1.17)	1.1 (0.86-1.41)	1.8 (1.17-2.77)	1.9 (1.42-2.53)	1.24 (0.78-1.98)	1.75 (1.37-2.24)
HCV genotype		P < 0.001	P < 0.001	P < 0.001	NA	NA	NA	NA
	1	1.0 (ref.)	1.0 (ref.)	1.0 (ref.)	1.0 (ref.)	1.0 (ref.)	1.0 (ref.)	1.0 (ref.)
	2	1.09 (0.86-1.39)	3.5 (2.38-5.15)	0.51 (0.36-0.71)	0.69 (0.47-1.02)	1.09 (0.85-1.4)	0.73 (0.51-1.05)	0.94 (0.76-1.15)
	3	1.47 (1.26-1.73)	2.5 (1.95-3.21)	1.57 (1.28-1.93)	1.4 (1.09-1.8)	1.4 (1.22-1.61)	0.93 (0.74-1.16)	1.26 (1.12-1.41)
	4	0.7 (0.56-0.87)	1.39 (0.9-2.13)	1.01 (0.75-1.37)	0.85 (0.57-1.27)	1.31 (1.07-1.61)	0.74 (0.51-1.07)	1.12 (0.94-1.33)
HIV status		P < 0.001	P = 0.66	P = 0.41	NA	NA	NA	NA
	Negative	1.0 (ref.)	1.0 (ref.)	1.0 (ref.)	1.0 (ref.)	1.0 (ref.)	1.0 (ref.)	1.0 (ref.)
	Positive	0.34 (0.26-0.45)	0.81 (0.46-1.44)	1.23 (0.85-1.79)	1.09 (0.72-1.66)	0.56 (0.41-0.75)	1.64 (1.21-2.22)	0.86 (0.7-1.06)
Chronic HBV infection		P = 0.89	P = 0.27	P = 0.13	NA	NA	NA	NA
	Negative	1.0 (ref.)	1.0 (ref.)	1.0 (ref.)	1.0 (ref.)	1.0 (ref.)	1.0 (ref.)	1.0 (ref.)
	Positive	0.83 (0.52-1.31)	0.96 (0.43-2.19)	1.74 (1.02-2.99)	1.29 (0.61-2.73)	0.66 (0.38-1.13)	1.46 (0.86-2.47)	0.86 (0.58-1.26)
Enrolment centre		P < 0.001	P < 0.001	P < 0.001	NA	NA	NA	NA
	Basel	1.0 (ref.)	1.0 (ref.)	1.0 (ref.)	1.0 (ref.)	1.0 (ref.)	1.0 (ref.)	1.0 (ref.)
	Bern	0.15 (0.11-0.21)	0.34 (0.23-0.51)	0.83 (0.59-1.16)	1.26 (0.78-2.06)	1.54 (1.17-2.01)	1.26 (0.86-1.85)	1.43 (1.15-1.78)
	Geneva	0.36 (0.24-0.53)	0.61 (0.38-0.98)	0.74 (0.48-1.13)	1.63 (0.95-2.81)	1.1 (0.78-1.56)	1.09 (0.68-1.73)	1.09 (0.83-1.44)
	Lausanne	0.35 (0.23-0.52)	0.48 (0.28-0.82)	1.24 (0.82-1.88)	1.31 (0.71-2.4)	1 (0.68-1.46)	0.7 (0.42-1.19)	0.89 (0.65-1.21)
	Lugano	0.36 (0.26-0.52)	0.66 (0.4-1.07)	0.66 (0.45-0.96)	1.18 (0.69-2.01)	1.38 (0.99-1.92)	1.18 (0.73-1.92)	1.27 (0.97-1.67)
	Neuch?tel	0.31 (0.21-0.44)	1 (0.6-1.66)	0.44 (0.28-0.68)	0.85 (0.47-1.57)	1.54 (1.13-2.1)	1.15 (0.71-1.87)	1.38 (1.06-1.78)
	St-Gall	0.37 (0.27-0.52)	1.21 (0.77-1.9)	0.46 (0.32-0.66)	1.49 (0.93-2.38)	1.64 (1.25-2.14)	1.35 (0.91-2.01)	1.48 (1.19-1.85)
	Z?rich	0.43 (0.31-0.58)	0.67 (0.46-0.97)	0.74 (0.53-1.02)	0.99 (0.62-1.59)	1.71 (1.33-2.2)	1.19 (0.82-1.73)	1.51 (1.23-1.86)
Cirrhotic at enrolment		P < 0.001	P = 0.036	X	X	NA	NA	NA
	No	1.0 (ref.)	1.0 (ref.)	X	X	1.0 (ref.)	1.0 (ref.)	1.0 (ref.)
	Yes	2.92 (2.4-3.56)	0.69 (0.53-0.9)	X	X	0.71 (0.58-0.88)	4.6 (3.77-5.61)	1.67 (1.46-1.9)
Treatment		X	X	X	NA	NA	NA	NA
	No treatment	X	X	X	1.0 (ref.)	1.0 (ref.)	1.0 (ref.)	1.0 (ref.)
	Treated with DAA	X	X	X	1.09 (0.77-1.55)	0.08 (0.04-0.13)	0.17 (0.1-0.27)	0.12 (0.08-0.16)
	Treated without DAA	X	X	X	0.95 (0.75-1.19)	1.02 (0.9-1.15)	0.7 (0.58-0.86)	0.92 (0.83-1.02)
Ever received DAA		X	P < 0.001	X	X	X	X	X
	No	X	1.0 (ref.)	X	X	X	X	X
	Yes	X	26.95 (17.61-41.26)	X	X	X	X	X

ATS= antiviral treatment status; SVR= sustained virologic response; CAE= cirrhosis at enrolment; IC= incident cirrhosis during follow-up; LTFU= loss to follow-up.
DAA= direct-acting antivirals.

Sensitivity analysis: comparison of persons by geographic origin, with removal of persons with missing values

		ATS	SVR	CAE	IC	LTFU	mortality	attrition
Geographic origin		P = 0.081	P = 0.14	P = 0.015	P = 0.81	P = 0.0039	P = 0.34	P = 0.16
	Switzerland	1.0 (ref.)	1.0 (ref.)	1.0 (ref.)	1.0 (ref.)	1.0 (ref.)	1.0 (ref.)	1.0 (ref.)
	Germany	0.85 (0.47-1.52)	1.12 (0.5-2.52)	1.03 (0.53-2)	0.5 (0.16-1.58)	1.79 (1.13-2.84)	0.63 (0.23-1.71)	1.31 (0.86-1.98)
	Italy	1.3 (0.93-1.82)	1.12 (0.71-1.75)	2.02 (1.43-2.87)	1.32 (0.85-2.05)	1.17 (0.86-1.59)	0.67 (0.44-1.01)	0.98 (0.77-1.26)
	Portugal	0.63 (0.37-1.05)	0.56 (0.25-1.28)	0.78 (0.37-1.66)	0.87 (0.35-2.2)	1.49 (0.95-2.34)	0.56 (0.17-1.78)	1.25 (0.82-1.89)
	Eastern Europe	0.67 (0.39-1.15)	1.61 (0.61-4.29)	0.97 (0.43-2.2)	1 (0.36-2.79)	1.64 (1.02-2.62)	0.64 (0.2-2.05)	1.42 (0.93-2.17)
	Southern Europe	1.08 (0.69-1.71)	2.12 (1.02-4.41)	1.47 (0.86-2.51)	1.21 (0.63-2.34)	1.79 (1.26-2.54)	1.04 (0.56-1.93)	1.46 (1.08-1.98)
	Western Europe	0.99 (0.66-1.48)	1.5 (0.79-2.84)	0.94 (0.55-1.59)	0.81 (0.43-1.55)	1.25 (0.88-1.78)	0.81 (0.46-1.44)	1.06 (0.78-1.43)
	Asia/Oceania	1.53 (0.85-2.75)	2.32 (1.09-4.95)	1.5 (0.79-2.83)	1.04 (0.42-2.61)	1.31 (0.87-1.98)	0.36 (0.11-1.15)	1.02 (0.7-1.5)
	Africa	0.79 (0.49-1.3)	1.14 (0.48-2.72)	1.29 (0.69-2.4)	1.39 (0.69-2.82)	0.93 (0.56-1.55)	0.84 (0.37-1.87)	0.89 (0.58-1.36)
	America	0.82 (0.45-1.48)	0.86 (0.34-2.13)	0.55 (0.21-1.49)	1.03 (0.41-2.58)	1.32 (0.76-2.28)	1.25 (0.46-3.45)	1.23 (0.76-1.98)
Gender		P < 0.001	P = 0.094	P < 0.001	P = 0.071	P = 0.75	P < 0.001	P = 0.12
	Female	1.0 (ref.)	1.0 (ref.)	1.0 (ref.)	1.0 (ref.)	1.0 (ref.)	1.0 (ref.)	1.0 (ref.)
	Male	1.37 (1.15-1.64)	0.89 (0.67-1.19)	1.52 (1.19-1.95)	1.16 (0.87-1.54)	0.97 (0.83-1.14)	1.22 (0.94-1.6)	1.03 (0.9-1.18)
Age (y)		P < 0.001	P = 0.04	P < 0.001	P < 0.001	P < 0.001	P < 0.001	P = 0.0018
	18-40	1.0 (ref.)	1.0 (ref.)	1.0 (ref.)	1.0 (ref.)	1.0 (ref.)	1.0 (ref.)	1.0 (ref.)
	41-60	1.64 (1.36-1.97)	0.76 (0.57-1.02)	5.26 (3.89-7.12)	2.69 (1.97-3.67)	0.73 (0.62-0.86)	1.64 (1.23-2.2)	0.85 (0.74-0.98)
	≥ 61	1.03 (0.71-1.49)	0.59 (0.34-1.02)	10.32 (6.57-16.21)	4.1 (2.37-7.08)	0.85 (0.57-1.26)	3.41 (2.14-5.44)	1.36 (1.02-1.8)
Education		P = 0.074	P = 0.06	P = 0.27	P = 0.44	P = 0.13	P = 0.11	P = 0.0061
	Low	0.93 (0.75-1.14)	0.84 (0.61-1.17)	1.07 (0.82-1.41)	1.16 (0.84-1.6)	1.17 (0.97-1.4)	1.19 (0.9-1.57)	1.15 (0.99-1.34)
	Middle	1.0 (ref.)	1.0 (ref.)	1.0 (ref.)	1.0 (ref.)	1.0 (ref.)	1.0 (ref.)	1.0 (ref.)
	High	0.96 (0.75-1.24)	1.33 (0.91-1.94)	1.05 (0.78-1.42)	1.19 (0.83-1.71)	1.05 (0.83-1.33)	1.02 (0.72-1.46)	1.04 (0.86-1.27)
Employment		P < 0.001	P = 0.092	P = 0.099	P = 0.28	P < 0.001	P < 0.001	P < 0.001
	Unempl.	1.0 (ref.)	1.0 (ref.)	1.0 (ref.)	1.0 (ref.)	1.0 (ref.)	1.0 (ref.)	1.0 (ref.)
	Working	1.34 (1.01-1.78)	0.72 (0.45-1.15)	1.09 (0.72-1.65)	0.83 (0.52-1.33)	0.6 (0.49-0.75)	0.69 (0.46-1.03)	0.62 (0.51-0.75)
	Inval.	1.08 (0.79-1.47)	0.49 (0.29-0.81)	1.29 (0.83-2.01)	0.87 (0.52-1.43)	0.61 (0.47-0.78)	1.24 (0.82-1.88)	0.76 (0.62-0.94)
(History of) injection drug use		P < 0.001	P = 0.012	P = 0.23	P = 0.17	P = 0.051	P = 0.2	P = 0.035
	Not user	1.0 (ref.)	1.0 (ref.)	1.0 (ref.)	1.0 (ref.)	1.0 (ref.)	1.0 (ref.)	1.0 (ref.)
	Former	0.59 (0.43-0.81)	1.08 (0.61-1.93)	0.61 (0.41-0.91)	0.95 (0.58-1.57)	1.27 (0.9-1.79)	1.19 (0.7-2.01)	1.17 (0.88-1.56)
	Current	0.62 (0.5-0.78)	1.22 (0.89-1.69)	0.53 (0.4-0.71)	0.72 (0.51-1.01)	1.34 (1.09-1.64)	0.86 (0.63-1.18)	1.15 (0.97-1.36)
Alcohol consumption		P = 0.037	P = 0.1	P < 0.001	P = 0.01	P = 0.13	P < 0.001	P = 0.0034
	Light	1.0 (ref.)	1.0 (ref.)	1.0 (ref.)	1.0 (ref.)	1.0 (ref.)	1.0 (ref.)	1.0 (ref.)
	Moderate	1.3 (1.02-1.66)	1.07 (0.75-1.54)	1.45 (1.06-1.98)	1.33 (0.93-1.92)	0.85 (0.68-1.06)	1.17 (0.82-1.67)	0.89 (0.74-1.07)
	Excessive	0.89 (0.72-1.11)	0.67 (0.47-0.94)	3.51 (2.65-4.66)	1.85 (1.3-2.62)	0.96 (0.79-1.17)	1.95 (1.44-2.62)	1.15 (0.98-1.35)
	Former	1.08 (0.77-1.52)	0.72 (0.41-1.28)	2.45 (1.6-3.77)	1.75 (1.05-2.91)	0.76 (0.55-1.06)	1.77 (1.17-2.7)	0.99 (0.76-1.27)
Time from diagnosis to enrolment (y)		P < 0.001	P < 0.001	P = 0.016	P = 0.072	P < 0.001	P = 0.3	P < 0.001
	0-2	1.0 (ref.)	1.0 (ref.)	1.0 (ref.)	1.0 (ref.)	1.0 (ref.)	1.0 (ref.)	1.0 (ref.)
	2-6	1.33 (1.08-1.64)	0.6 (0.43-0.84)	0.99 (0.74-1.33)	1.13 (0.8-1.59)	0.77 (0.64-0.92)	0.78 (0.58-1.05)	0.78 (0.66-0.91)
	6-10	1.76 (1.38-2.24)	0.69 (0.48-0.98)	1.27 (0.94-1.71)	1.43 (1-2.05)	0.77 (0.63-0.96)	1.04 (0.77-1.41)	0.83 (0.7-0.99)
	10+	1.58 (1.23-2.04)	0.58 (0.4-0.84)	1.47 (1.09-1.98)	1.47 (1.01-2.14)	0.58 (0.44-0.76)	0.85 (0.6-1.2)	0.66 (0.54-0.82)
Calendar year of enrolment		P < 0.001	P = 0.16	P = 0.55	P = 0.082	P < 0.001	P = 0.71	P < 0.001
	2000-2003	1.0 (ref.)	1.0 (ref.)	1.0 (ref.)	1.0 (ref.)	1.0 (ref.)	1.0 (ref.)	1.0 (ref.)
	2004-2007	0.77 (0.63-0.94)	0.75 (0.56-1.01)	1.18 (0.91-1.53)	1.27 (0.95-1.71)	1.6 (1.36-1.88)	1.13 (0.87-1.46)	1.46 (1.27-1.67)
	2008-2011	0.79 (0.58-1.09)	0.93 (0.54-1.61)	0.93 (0.62-1.4)	1.08 (0.62-1.86)	2.47 (1.89-3.24)	1.16 (0.7-1.9)	2.11 (1.66-2.67)
	2012-2017	0.47 (0.34-0.63)	0.58 (0.29-1.16)	1.08 (0.74-1.57)	2.04 (1.12-3.72)	1.64 (1.02-2.65)	0.83 (0.34-2.04)	1.4 (0.92-2.14)
HCV genotype		P < 0.001	P < 0.001	P < 0.001	P = 0.021	P < 0.001	P < 0.001	P < 0.001
	1	1.0 (ref.)	1.0 (ref.)	1.0 (ref.)	1.0 (ref.)	1.0 (ref.)	1.0 (ref.)	1.0 (ref.)
	2	0.82 (0.58-1.17)	4.16 (2.51-6.89)	0.4 (0.25-0.65)	0.64 (0.39-1.06)	0.94 (0.65-1.35)	0.49 (0.28-0.84)	0.72 (0.53-0.97)
	3	1.33 (1.1-1.62)	2.98 (2.22-4.02)	1.6 (1.25-2.06)	1.38 (1.02-1.85)	1.44 (1.22-1.7)	0.87 (0.66-1.13)	1.26 (1.1-1.45)
	4	0.67 (0.51-0.88)	1.68 (1.01-2.78)	0.99 (0.68-1.44)	0.76 (0.47-1.24)	1.17 (0.91-1.51)	0.69 (0.46-1.05)	1.01 (0.82-1.26)
HIV status		P < 0.001	P = 0.25	P = 0.43	P = 0.16	P = 0.0013	P < 0.001	P = 0.77
	Negative	1.0 (ref.)	1.0 (ref.)	1.0 (ref.)	1.0 (ref.)	1.0 (ref.)	1.0 (ref.)	1.0 (ref.)
	Positive	0.28 (0.2-0.38)	0.62 (0.33-1.19)	1.29 (0.83-2)	1.31 (0.81-2.11)	0.6 (0.43-0.85)	2 (1.42-2.81)	1 (0.79-1.26)
Chronic HBV infection		P = 0.9	P = 0.98	P = 0.15	P = 0.75	P = 0.052	P = 0.38	P = 0.33
	Negative	1.0 (ref.)	1.0 (ref.)	1.0 (ref.)	1.0 (ref.)	1.0 (ref.)	1.0 (ref.)	1.0 (ref.)
	Positive	0.95 (0.51-1.76)	0.94 (0.37-2.39)	1.71 (0.87-3.35)	0.81 (0.3-2.21)	0.55 (0.26-1.17)	1.4 (0.73-2.7)	0.85 (0.53-1.39)
Enrolment centre		P < 0.001	P < 0.001	P < 0.001	P = 0.41	P = 0.0013	P = 0.026	P < 0.001
	Basel	1.0 (ref.)	1.0 (ref.)	1.0 (ref.)	1.0 (ref.)	1.0 (ref.)	1.0 (ref.)	1.0 (ref.)
	Bern	0.14 (0.09-0.23)	0.33 (0.2-0.55)	0.87 (0.55-1.37)	1.06 (0.59-1.9)	1.59 (1.12-2.25)	1.09 (0.69-1.73)	1.42 (1.07-1.87)
	Geneva	0.31 (0.18-0.53)	0.85 (0.45-1.6)	0.6 (0.32-1.1)	1.48 (0.76-2.88)	1.15 (0.72-1.84)	1.09 (0.61-1.96)	1.14 (0.79-1.64)
	Lausanne	0.23 (0.14-0.4)	0.67 (0.35-1.29)	1.37 (0.79-2.38)	1.2 (0.59-2.44)	1.14 (0.71-1.82)	0.5 (0.25-0.97)	0.87 (0.59-1.27)
	Lugano	0.26 (0.16-0.44)	0.67 (0.34-1.34)	0.49 (0.27-0.89)	1.08 (0.55-2.14)	1.01 (0.62-1.63)	0.73 (0.35-1.53)	0.86 (0.57-1.28)
	Neuchâtel	0.27 (0.17-0.43)	1.08 (0.6-1.93)	0.55 (0.32-0.94)	0.74 (0.37-1.5)	1.65 (1.12-2.42)	0.88 (0.49-1.57)	1.33 (0.97-1.83)
	St-Gall	0.35 (0.23-0.54)	1.18 (0.69-2)	0.54 (0.34-0.87)	1.34 (0.77-2.35)	1.67 (1.18-2.37)	1.02 (0.64-1.64)	1.39 (1.05-1.83)
	Zürich	0.4 (0.26-0.61)	0.67 (0.43-1.05)	1.09 (0.72-1.66)	1.03 (0.59-1.79)	1.74 (1.26-2.41)	0.98 (0.64-1.51)	1.42 (1.1-1.84)
Cirrhotic at enrolment		P < 0.001	P = 0.015	X	X	P < 0.001	P < 0.001	P < 0.001
	No	1.0 (ref.)	1.0 (ref.)	X	X	1.0 (ref.)	1.0 (ref.)	1.0 (ref.)
	Yes	2.83 (2.14-3.74)	0.6 (0.43-0.84)	X	X	0.7 (0.53-0.92)	4.62 (3.57-5.96)	1.67 (1.41-1.98)
Treatment		X	X	X	P = 0.55	P < 0.001	P < 0.001	P < 0.001
	No treatment	X	X	X	1.0 (ref.)	1.0 (ref.)	1.0 (ref.)	1.0 (ref.)
	Treated with DAA	X	X	X	0.72 (0.43-1.22)	0.03 (0.01-0.1)	0.19 (0.1-0.36)	0.1 (0.06-0.16)
	Treated without DAA	X	X	X	0.9 (0.68-1.19)	1.03 (0.87-1.21)	0.72 (0.56-0.92)	0.93 (0.81-1.06)
Ever received DAA		X	P < 0.001	X	X	X	X	X
	No	X	1.0 (ref.)	X	X	X	X	X
	Yes	X	26.02 (15.07-44.9)	X	X	X	X	X

ATS= antiviral treatment status; SVR= sustained virologic response; CAE= cirrhosis at enrolment; IC= incident cirrhosis during follow-up; LTFU= loss to follow-up. DAA= direct-acting antivirals.
